# Bridging data and discovery: a survey on knowledge graphs in AI for science

**DOI:** 10.1093/nsr/nwag140

**Published:** 2026-03-05

**Authors:** Keyan Ding, Zhihui Zhu, Yuqi Tang, Kehua Feng, Xiang Zhuang, Hongwei Wang, Yi Yang, Huifang Du, Zhangkai Ni, Shiqi Wang, Xiaohui Fan, Huabin Xing, Lei Bai, Qi Liu, Haofen Wang, Qiang Zhang, Huajun Chen

**Affiliations:** College of Computer Science and Technology, Zhejiang University, Hangzhou 310027, China; ZJU-Hangzhou Global Scientific and Technological Innovation Center, Zhejiang University, Hangzhou 311200, China; ZJU-Hangzhou Global Scientific and Technological Innovation Center, Zhejiang University, Hangzhou 311200, China; ZJU-UIUC Institute, Zhejiang University, Haining 314400, China; College of Computer Science and Technology, Zhejiang University, Hangzhou 310027, China; College of Computer Science and Technology, Zhejiang University, Hangzhou 310027, China; Shanghai Artificial Intelligence Laboratory, Shanghai 200232, China; ZJU-UIUC Institute, Zhejiang University, Haining 314400, China; ZJU-Hangzhou Global Scientific and Technological Innovation Center, Zhejiang University, Hangzhou 311200, China; College of Design and Innovation, Tongji University, Shanghai 200092, China; College of Computer Science and Technology, Tongji University, Shanghai 201804, China; Department of Computer Science, City University of Hong Kong, Hong Kong 999077, China; College of Pharmaceutical Sciences, Zhejiang University, Hangzhou 310058, China; College of Chemical and Biological Engineering, Zhejiang University, Hangzhou 310027, China; Shanghai Artificial Intelligence Laboratory, Shanghai 200232, China; School of Life Sciences and Technology, Tongji University, Shanghai 200092, China; College of Design and Innovation, Tongji University, Shanghai 200092, China; ZJU-Hangzhou Global Scientific and Technological Innovation Center, Zhejiang University, Hangzhou 311200, China; ZJU-UIUC Institute, Zhejiang University, Haining 314400, China; College of Computer Science and Technology, Zhejiang University, Hangzhou 310027, China; ZJU-Hangzhou Global Scientific and Technological Innovation Center, Zhejiang University, Hangzhou 311200, China

**Keywords:** scientific knowledge graph, AI for science, knowledge-driven framework, autonomous scientific discovery

## Abstract

Knowledge graphs have emerged as a powerful paradigm for structuring, organizing and reasoning over complex scientific knowledge, and are increasingly recognized as catalysts for accelerating AI for science. This study provides a comprehensive survey of scientific knowledge graphs (SciKGs), covering their construction methodologies and diverse applications across biology, chemistry and materials science. We examine how SciKGs support tasks such as drug development, omics analysis, reaction prediction and materials design, and highlight how the synergistic integration of SciKGs and large language models (LLMs) forms a knowledge- and language-driven framework for scientific discovery, in which SciKGs serve as the foundational knowledge infrastructure and LLMs act as dynamic semantic engines. We further identify key challenges and outline emerging opportunities for building auditable, interoperable and self-evolving SciKGs. Looking forward, we envision a new generation of SciKG-centered ecosystems where self-updating graphs, co-evolving with LLMs and embodied within AI scientists, become core infrastructures that autonomously drive, verify and accelerate scientific discovery.

## Teaser

Knowledge graphs are becoming core infrastructures for AI-driven scientific discovery. This work reviews how they are built and used across biology, chemistry, and materials science.

## INTRODUCTION

Scientific discovery is undergoing a paradigm shift from intuition-driven exploration to data-intensive, AI-powered inference. The deluge of high-throughput experiments, large-scale simulations and multi-modal sensing technologies has generated unprecedented volumes of heterogeneous, complex data across biology, chemistry and materials science [1,2]. Yet, this data explosion has not been matched by a corresponding leap in our ability to synthesize, contextualize and reason over it. Fragmentation across formats, terminologies and domains leaves vast reservoirs of scientific knowledge underutilized—a ‘knowledge gap’ that threatens to widen as data generation outpaces human interpretability [3]. Addressing this challenge requires computational frameworks capable of unifying, representing and reasoning over large-scale knowledge.

Knowledge graphs (KGs) have emerged as a powerful paradigm for organizing structured information by representing entities and their relations in a machine-interpretable form [4,5]. Generally, a KG can be defined as a directed, labeled graph in which nodes represent entities and edges denote semantic relations among them. In scientific domains, KGs provide a unifying representation of diverse entities, such as genes, proteins, diseases, chemical compounds and materials, capturing their intricate relationships across experimental and computational contexts [6,7]. Over the past decades, scientific knowledge graphs (SciKGs) have been applied to diverse problems such as drug repurposing, multi-omics

analysis, chemical reaction modeling and materials design [8–10], demonstrating their potential as engines of discovery.

However, constructing SciKGs remains technically demanding. Entity and relation extraction, ontology alignment and knowledge integration must contend with unstructured scientific texts, inconsistent terminologies and rapidly evolving knowledge. Traditional rule-based or ontology-driven approaches provide valuable structure but often lack scalability and adaptability in the face of scientific data complexity [3,11]. The integration of artificial intelligence (AI) techniques, particularly large language models (LLMs) [12], has begun to transform this landscape. LLMs can automate knowledge extraction from unstructured literature, enrich semantic representations and predict missing links within graphs [13,14]. Conversely, SciKGs provide structured grounding for LLMs, improving factual reliability, contextual reasoning and reducing hallucinations in generative scientific tasks [15,16]. This bidirectional synergy between SciKGs and LLMs is opening new opportunities for AI-driven scientific reasoning, hypothesis generation and decision support.

Despite growing interest, most surveys to date have concentrated on general-purpose KGs [17–19], providing valuable overviews of graph construction techniques and applications but offering limited insight into the unique demands of scientific domains. Existing reviews of SciKGs [11,20] remain fragmented, often narrowing their scope to a single scientific field such as biomedicine. Moreover, they rarely explore the integration of SciKGs with LLMs, neglecting one of the most transformative developments in the field. What is still lacking is a unified, cross-disciplinary perspective that captures the full landscape of SciKGs (from construction and integration to application and evolution) and highlights their symbiosis with LLMs as a catalyst for accelerating discovery.

**Figure 1.**
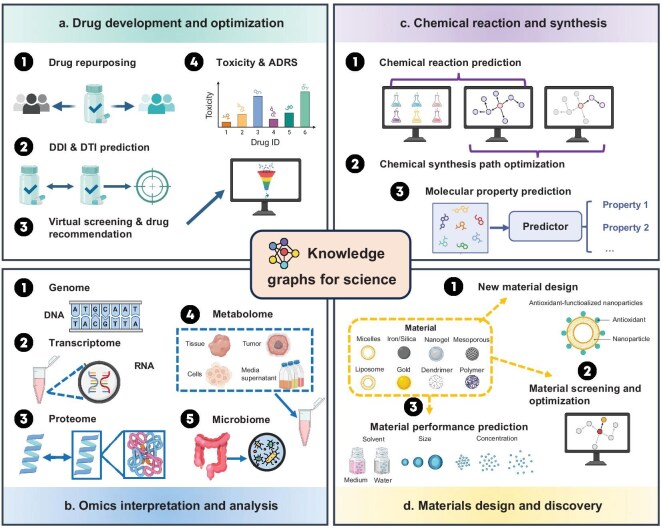
Overview of the research scope in this survey, covering four fundamental scientific tasks in biology, chemistry and materials science: (a) drug development and optimization, (b) omics interpretation and analysis, (c) chemical reaction and synthesis, and (d) materials design and discovery. Icons from BioRender.com.

In this study, we fill this gap and provide a comprehensive survey of KGs in the fundamental scientific domains, particularly focusing on biology, chemistry and materials science (Fig. 1). Specifically, we make four distinctive contributions. First, we systematically examine how SciKGs are constructed and applied across diverse scientific domains, highlighting their roles in advancing drug development, omics analysis, chemical synthesis and materials discovery. Second, we place particular emphasis on the integration of SciKGs with LLMs for scientific discovery, showing how this emerging synergy opens new opportunities for knowledge extraction, reasoning and generation. Third, we highlight unresolved challenges and propose concrete research directions to guide the development of next-generation knowledge discovery systems in the LLM era. Fourth, we establish and actively maintain a curated, open-access repository for SciKGs at GitHub (https://github.com/hicai-zju/scikgs), which provides up-to-date resources including literature, datasets and software. Together, these contributions establish this work as a comprehensive, living reference and a strategic roadmap for advancing scientific knowledge graphs in the era of AI-driven discovery.

Ultimately, this review aims to address a core question: how can knowledge graphs serve as the structured knowledge infrastructure for AI for science, and how can SciKGs and LLMs synergize to enable future autonomous scientific discovery? By bridging the precision of symbolic knowledge with the generative power of large models, we argue that SciKGs are not merely data repositories but the essential deterministic substrate required to ground, validate and accelerate the next generation of scientific intelligence.

**Figure 2.**
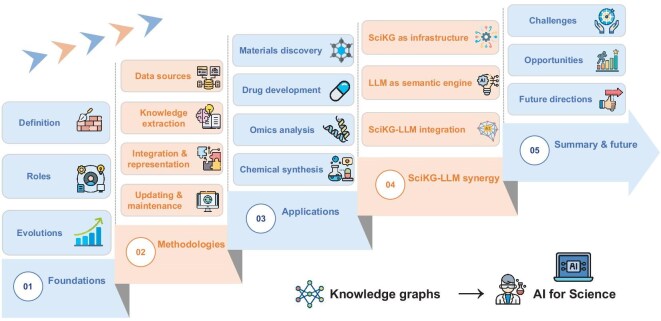
Structure of the survey. Our review is structured around the lifecycle of SciKGs: from their conceptual foundation and construction methodologies, to their applications and synergistic integration with LLMs for discovery, culminating in challenges, opportunities and future directions that envision SciKGs as engines for autonomous scientific discovery.

The remainder of this review provides a roadmap for understanding, constructing and leveraging SciKGs to accelerate AI for science (Fig. 2). We first lay the conceptual foundation by defining SciKGs, outlining their key roles in organizing and reasoning over scientific knowledge, and tracing their historical evolution. We then guide readers through the construction process, detailing strategies for integrating heterogeneous data, extracting entities and relations, aligning ontologies and curating high-quality graphs. Next, we chart the diverse domains where SciKGs are applied, with illustrative examples in biology, chemistry and materials science, showing how these graphs enable interpretation, prediction and generation. We further discuss how SciKGs can be combined with large language models to accelerate scientific discovery, emphasizing their complementary roles in knowledge grounding and reasoning. Finally, we outline the key challenges and opportunities that define the next stage in SciKG development, offering a forward-looking perspective on building robust knowledge infrastructures for LLM-driven autonomous AI systems.

## CONCEPTUAL FOUNDATIONS OF SCIENTIFIC KNOWLEDGE GRAPHS

Scientific knowledge graphs provide a structured, semantically rich and computable representation of scientific entities, their relationships and contextual information across diverse disciplines. Unlike general-purpose knowledge graphs that prioritize broad coverage and common-sense reasoning, SciKGs are purpose-built to encode domain-specific semantics, causal relationships and contextual constraints inherent to scientific inquiry. In this section, we introduce their definitions, roles and evolution in scientific discovery, laying the conceptual foundation for subsequent discussions on construction methodologies and applications.

### Definitions

Formally, a SciKG can be defined as a directed, labeled graph $G = (V, E)$, where each node $v \in V$ represents a scientific entity (e.g. a gene, protein, compound, reaction or material), and each edge $e \in E$ denotes a semantic relation between entities (e.g. activation, inhibition, binding, catalysis or synthesis). In addition to structural connectivity, nodes and edges are often enriched with metadata such as provenance, experimental conditions, quantitative attributes and links to external databases or literature references. This multi-layered representation transforms raw scientific data into an interconnected knowledge fabric that supports both human interpretability and automated reasoning. Moreover, SciKGs increasingly incorporate temporal, contextual and multi-modal dimensions. Temporal edges encode the evolution of knowledge over time, capturing how hypotheses, measurements and discoveries emerge or are refuted. Contextual layers specify experimental settings, material compositions or biological environments in which relations hold. Multi-modal extensions integrate textual, numerical and visual modalities—e.g. linking microscopy images or spectroscopy spectra to molecular entities—creating a richer and more expressive knowledge representation suitable for data-intensive science. Crucially, it is important to distinguish SciKGs from scientific data graphs. While data graphs (e.g. crystal-structure graphs representing atomic connectivity, or raw protein-interaction networks based on correlations) focus on the geometric or topological structure of specific data samples, SciKGs are fundamentally defined by their semantic backbone rooted in domain ontologies. In a multi-modal SciKG, numerical, visual and temporal data do not replace the semantic graph but serve as multi-modal attributes or grounding evidence linked to specific entities, thereby enriching the symbolic knowledge with dense, computable representations.

### Roles

SciKGs serve as a foundational infrastructure that bridges data, knowledge and intelligence in scientific discovery. Their roles can be categorized along four axes.

1. *Knowledge organization.* SciKGs unify heterogeneous data sources, spanning biological sequences, chemical structures, material properties and experimental records, under a consistent semantic schema. This unification mitigates data fragmentation, improves interoperability and provides researchers with a single point of access for querying and integrating diverse knowledge.
2. *Knowledge embedding.* SciKGs provide a scaffold for learning contextualized embeddings of scientific entities. Through knowledge graph embedding (KGE) approaches [20,21], entities and relations are projected into continuous vector spaces in which geometric proximity encodes semantic relatedness. These representations enrich downstream tasks such as drug–target prediction, materials property estimation or pathway inference by injecting structured scientific priors into model learning.
3. *Knowledge inference.* By encoding relational dependencies, SciKGs enable various forms of reasoning such as link prediction, causal inference and hypothesis generation. Graph algorithms and embedding-based approaches [22] allow the prediction of novel interactions (e.g. drug–target binding, gene–disease associations or reaction pathways) that may not be explicitly observed in experimental data.
4. *Knowledge interpretability.* Unlike black-box predictive models, SciKGs preserve explicit semantic relationships and traceable provenance information [23]. This transparency allows scientists to validate model predictions, interpret causal chains and connect inferred results back to experimental evidence or literature sources, fostering trust and accountability in AI-driven discovery.

### Evolutions

**Figure 3.**
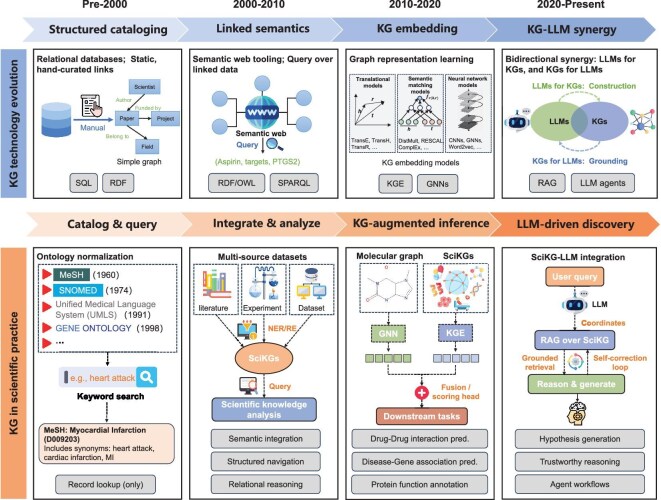
The co-evolution of knowledge graph technologies and their scientific practices. The technological evolution of KGs (top) has continually enabled new paradigms in SciKG applications (bottom). This progression has moved from static cataloging and manual integration to machine-learning-driven inference, culminating in the current era of bidirectional synergy between LLMs and KGs. This synergy, leveraging tools such as RAG and AI agents, transforms SciKGs from static repositories into dynamic engines for generative scientific discovery. SQL, Structured Query Language; RDF, Resource Description Framework; OWL, Web Ontology Language; SPARQL, SPARQL protocol and RDF query language; GNN, graph neural network; KGE, knowledge graph embedding; RAG, retrieval-augmented generation.

The development of SciKGs has undergone several transformative phases (Fig. 3), reflecting the co-evolution between knowledge-representation technologies and scientific practices. Here we identify four key phases.

1. *Cataloging era (pre-2000s).* Early efforts focused on structured databases and controlled vocabularies (e.g. GenBank [24], PDB [25]). Knowledge was stored in relational tables with limited semantic expressivity, primarily supporting lookup and retrieval rather than reasoning.
2. *Semantic web era (2000s–2010s).* The introduction of the Resource Description Framework (RDF), Web Ontology Language (OWL) and SPARQL enabled the formal representation of scientific entities and relationships, giving rise to semantically interoperable knowledge systems. Initiatives such as Bio2RDF [26] and the Open Biological and Biomedical Ontology (OBO) Foundry [27] exemplified this era, promoting cross-database reasoning and federated query capabilities.
3. *Machine learning era (2010s–2020s).* With the emergence of graph embeddings and graph neural networks, SciKGs evolved into predictive engines capable of inferring new links and patterns from existing knowledge. Representation learning (e.g. TransE [28], GraphSAGE [29]) bridged the gap between symbolic knowledge and numerical computation, unlocking applications in drug repurposing, reaction prediction and materials property estimation.
4. *Large language model era (2020s–present).* The integration of large language models has catalyzed a new paradigm. LLMs automate KG construction from literature (e.g. AutoKG [30]), generate hypotheses grounded in SciKGs (e.g. SciAgents [31]) and serve as natural language interfaces for complex queries (e.g. DDI-GPT [32]). Conversely, SciKGs mitigate LLM hallucinations via retrieval-augmented generation (RAG) and provide structured constraints for scientific plausibility. This bidirectional synergy transforms SciKGs from static knowledge storage to intelligent infrastructures.

Overall, the conceptual evolution of SciKGs mirrors the broader transformation of scientific inquiry: from static cataloging to semantic reasoning, and now toward autonomous, knowledge-augmented discovery. By bridging structured knowledge and generative intelligence, SciKGs lay the foundation for a new era of AI-driven scientific discovery.

## METHODOLOGIES FOR CONSTRUCTING SCIENTIFIC KNOWLEDGE GRAPHS

**Figure 4.**
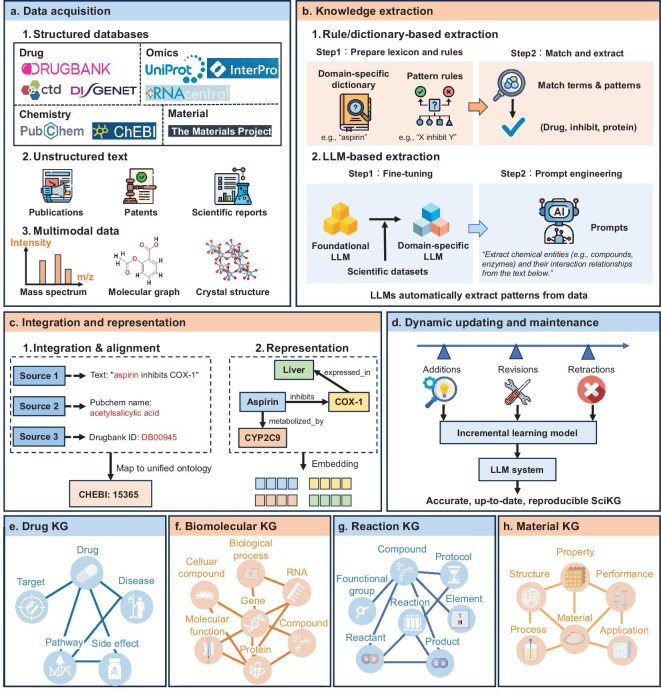
Construction and maintenance of SciKGs. (a) The foundation of SciKG construction involves integrating diverse data sources, including structured databases, unstructured text and multi-modal data. (b) Two main approaches for extracting entities and relations from the acquired data are illustrated: rule- or dictionary-based extraction, which relies on predefined lexicons and rules, and LLM-based extraction, which involves fine-tuning on scientific datasets and prompt engineering. (c) Ontology alignment integrates diverse representations of the same entity (e.g. aspirin), followed by graph embedding into a continuous vector space. (d) Dynamic updating through incremental learning and LLM-driven error correction ensures SciKGs remain accurate and up to date. (e–h) Subfigures illustrate representative examples of specialized knowledge graphs for drugs, omics, chemicals and materials, respectively. Icons from BioRender.com.

The construction of SciKGs is a multi-stage process that involves integrating heterogeneous data sources, extracting entities and relations, aligning knowledge with existing ontologies and ensuring the dynamic maintainability of the resulting graphs (Fig. 4). Unlike general-purpose knowledge graphs, SciKGs face the additional complexity of representing domain-specific entities such as genes, proteins and molecules, which often require fine-grained semantic modeling and contextual reasoning. In this section, we briefly review the major aspects of SciKG construction, including data sources, extraction techniques, integration strategies and maintenance approaches. We also highlight emerging trends in multi-modal SciKGs, which are increasingly critical for capturing the full complexity of scientific data. Tables S1 and S2 summarize commonly used resources (including databases, software and tools) for SciKG construction and management.

### Data sources

SciKGs are built from a wide range of scientific data sources, which can be broadly categorized into structured databases, unstructured text and multi-modal repositories.

Structured data sources form the semantic backbone of most domain-specific graphs. Well-curated repositories such as PubChem [33], UniProt [34], the Protein Data Bank (PDB) [25] and the Materials Project [35] provide standardized, machine-readable annotations of molecular structures, protein interactions, crystal lattices and thermodynamic properties. These resources are typically developed under community-endorsed standards and employ persistent identifiers (e.g. DOIs, InChI or UniProt accessions), ensuring interoperability and reproducibility. As such, they serve as stable and verifiable foundations for constructing large-scale SciKGs.

Unstructured textual sources (including scientific papers, patents, laboratory notebooks and experimental reports) represent the most abundant yet least structured form of scientific knowledge. Massive text corpora such as PubMed, arXiv and the USPTO database collectively encode millions of entities, relations and claims expressed in natural language. Extracting structured knowledge from these heterogeneous materials requires advanced natural language processing (NLP) pipelines, encompassing named-entity recognition, dependency parsing, event extraction and relation detection.

Multi-modal data sources are increasingly indispensable for capturing the quantitative and contextual complexity of modern science. These encompass a wide spectrum of experimental and computational modalities: omics profiles (e.g. RNA-seq, proteomics, metabolomics) that quantify molecular abundance; imaging data such as electron microscopy, fluorescence microscopy or X-ray diffraction patterns; spectroscopic signals (e.g. NMR) that encode molecular fingerprints; computational simulations, including molecular dynamics trajectories or density functional theory outputs; time-series experimental measurements, such as thermal degradation curves or electrochemical cycling data. The integration of these modalities enables multi-modal SciKGs, which enrich symbolic triples by anchoring numerical, visual, temporal and topological evidence as attributes of semantic entities. Crucially, these multi-modal data serve as grounding context rather than replacing the ontological backbone of the SciKG.

### Knowledge extraction

Extracting entities, relations and scientific events from heterogeneous data remains one of the core challenges in constructing SciKGs. Unlike general-purpose information extraction, scientific data involve highly specialized terminologies, nested relationships and evolving conceptual frameworks that demand fine-grained semantic understanding and context-aware reasoning. Consequently, the design of SciKG extraction pipelines must reconcile three often conflicting requirements: precision, scalability and adaptability to emerging knowledge.

Traditional rule- and ontology-based methods [36,37] represent the earliest efforts toward structured knowledge extraction in domains such as biomedicine and chemistry. These systems leverage domain-specific dictionaries, handcrafted rules and curated ontologies (e.g. Gene Ontology) to identify entities and align them with controlled vocabularies. Their advantages lie in interpretability, reproducibility and high precision within well-defined subdomains. However, they suffer from limited scalability, domain transferability and poor adaptability to newly emerging concepts.

Data-driven NLP approaches [38,39] have since transformed the field by enabling automated, large-scale extraction from unstructured scientific text. Techniques such as named-entity recognition, relation extraction and event detection have been adapted to domain corpora using models like SciBERT [40] and domain-tuned transformers. These models outperform rule-based systems in recall and generalization, particularly when combined with weak supervision or self-training. More recently, LLMs have revolutionized scientific information extraction [41]. Through few-shot prompting and task-specific fine-tuning, LLMs can recognize novel entities, infer implicit relations and even generate structured hypotheses from textual evidence, bridging the gap between symbolic extraction and conceptual understanding.

Hybrid and semi-automated pipelines [42] are emerging as practical solutions for reconciling the conflicting requirements of precision, scalability and adaptability (see Table S3 for a detailed comparative analysis of these paradigms). By combining the domain precision of ontology-based methods with the generalization of neural models, these frameworks address the intrinsic limitations of individual approaches. For instance, rule-based prefilters can identify candidate entities with high confidence, which are then refined using transformer-based relation classifiers. Conversely, neural models can suggest new candidate relations or ontological extensions, which are validated against existing controlled vocabularies. This synergy reduces both annotation costs and error propagation, while maintaining interpretability—an essential requirement for scientific credibility and trustworthiness.

### Integration and representation

After the extraction of entities and relations, a critical challenge lies in transforming fragmented knowledge into a coherent and semantically consistent structure. Integration and representation serve as the foundation for building interoperable and computationally tractable SciKGs. To achieve semantic consistency across heterogeneous data sources, ontology-alignment and schema-matching techniques [43] are widely employed. These methods harmonize terminologies, reconcile conceptual discrepancies and enable cross-domain reasoning. Recent frameworks increasingly adopt federated ontology mapping [44] and probabilistic schema alignment [45], which tolerate terminological uncertainty and facilitate large-scale integration across biomedical, chemical and materials databases.

After achieving semantic interoperability, the next step is to encode the integrated graph into representations that support computation and reasoning. Representation learning has emerged as a key paradigm for capturing the relational and structural regularities within scientific knowledge. Graph-based embedding methods [20] project nodes, relations and subgraphs into continuous vector spaces, preserving both topological proximity and semantic dependencies. These representations bridge the gap between symbolic integration and data-driven inference, enabling efficient similarity computation, link prediction and hypothesis generation. However, the choice of representation architecture significantly impacts downstream utility. Shallow KGEs (e.g. TransE) are computationally efficient and effective for massive, relation-dense networks (e.g. drug interactions) but often struggle with inductive inference on unseen entities. In contrast, GNNs aggregate neighborhood features to support inductive reasoning, making them superior for structure-rich domains such as molecular chemistry. Recently, LLM-based encodings have emerged to capture fine-grained semantic nuance from unstructured text, offering strong zero-shot capabilities albeit with higher inference latency compared with structural methods.

Moreover, scientific knowledge is often multi-modal, with entity descriptions appearing in various forms such as text, microscopy images, molecular graphs or time-series experimental measurements. To capture these characteristics, methods such as cross-modal embedding [46] have been introduced to model interactions across heterogeneous data types effectively. These methods align heterogeneous modalities into shared latent spaces, enabling unified reasoning over textual, visual and structural data, and even support temporal inference over evolving scientific processes.

### Updating and maintenance

Scientific knowledge is continuously evolving, with new discoveries, revised findings and retracted claims constantly reshaping the landscape. Thus, SciKGs must be designed as dynamic and adaptive systems rather than static repositories. Incremental learning approaches [47] allow the seamless integration of new data while minimizing catastrophic forgetting of prior knowledge. Beyond algorithmic updating, community and human-in-the-loop mechanisms are essential for maintaining trustworthiness. Crowdsourced and expert-driven curation initiatives, exemplified by biomedical resources such as UniProt and the Gene Ontology, demonstrate that combining automated extraction with domain expertise yields more accurate and interpretable updates.

Meanwhile, automated verification and maintenance pipelines are increasingly powered by LLMs agents [48]. These agents can detect inconsistencies, contradictions and obsolete links by comparing textual evidence, citation networks or temporal trends. Automated correction mechanisms then propagate verified updates across dependent nodes and relations, improving graph coherence and reducing cumulative error. Integration with provenance metadata and version-control frameworks further ensures reproducibility and traceability, which are core requirements for scientific accountability.

### Evaluation

The utility of a SciKG fundamentally depends on its quality and reliability. Evaluation strategies typically span three levels of granularity to ensure rigorous validation (see Table S4 for a comprehensive taxonomy of metrics and benchmarks).

1. *Component-level evaluation.* This dimension focuses on the fidelity of the construction pipeline. The accuracy of entity and relation extraction is typically assessed via precision, recall and F1 scores against gold-standard corpora, while ontology-alignment correctness is evaluated using reference mappings to ensure semantic consistency across heterogeneous sources.
2. *Graph-level representation.* Beyond individual facts, the structural adequacy of the KG is critical for downstream inference. Metrics such as graph density and connectivity are analyzed alongside embedding quality. Link-prediction benchmarks are widely employed to measure how well the graph captures latent scientific associations using metrics like mean reciprocal rank (MRR) and Hits@k.
3. *Trustworthiness and utility.* In the context of AI for science, evaluation extends to trustworthiness, specifically provenance coverage and temporal consistency. Ultimately, the representational adequacy of a SciKG is validated extrinsically by its performance uplift in specific scientific tasks, such as drug repurposing or material-property prediction.

### Summary and prospects

The construction of SciKGs is no longer a one-time curation effort but an ongoing, adaptive process that bridges structured databases, unstructured literature and rich multi-modal evidence. The rise of multi-modal SciKGs marks a pivotal shift toward more holistic, quantitative and interpretable scientific knowledge infrastructures. The integration of LLMs and advanced multi-modal approaches has further accelerated KG construction by enabling automated entity recognition, relation extraction and error correction at unprecedented scale and speed. Future directions include (i) standardizing multi-modal KG schemas across domains; (ii) enabling real-time KG evolution via autonomous LLM agents and (iii) fostering open, community-governed platforms for collaborative KG curation. As SciKGs continue to evolve from static repositories to dynamic and adaptive knowledge infrastructures, these advances will directly improve the efficiency, accuracy and comprehensiveness of KG construction, laying the foundation for more reliable and integrative scientific knowledge representation.

## APPLICATIONS OF SCIENTIFIC KNOWLEDGE GRAPHS

**Figure 5.**
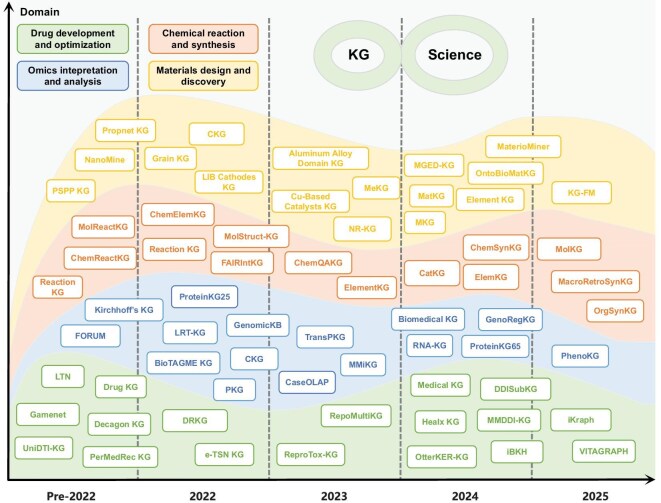
A taxonomy of existing SciKGs and their applications in science. More details of these SciKGs are presented in Tables S5–S8. An extended comparison of SciKGs across six dimensions (construction methodologies, benchmarking performance, practical usability, scalability, reproducibility and data quality) is available at our open-access repository: https://github.com/hicai-zju/scikgs.

Knowledge graphs organize multi-source scientific knowledge into linked, computable structures that support data-driven reasoning in complex scientific problems. In this section, we highlight representative applications of SciKGs in four domain tasks: drug development and optimization, omics interpretation and analysis, chemical reaction and synthesis, and materials design and discovery (Fig. 5). These applications demonstrate how SciKGs serve as engines of scientific discovery by facilitating inference, prediction and decision-making processes.

### Drug development and optimization

The drug development process is prone to high attrition due to fragmented data, intricate biological mechanisms and limited translational fidelity between preclinical and clinical phases. KGs address these challenges by integrating molecular, phenotypic, clinical and literature-based information into semantically rich networks, enabling coherent reasoning across drugs, targets and diseases. In drug repurposing, KGs uncover non-obvious drug–disease relationships by synthesizing multi-omics, literature and pathway data, supporting mechanistic inference and rapid candidate identification for rare diseases and epidemic contexts [49,50]. For instance, the TxGNN model [49], pre-trained on a large-scale medical KG, enables zero-shot prediction across more than 17 000 diseases, demonstrating how KG-derived representations can generalize to diseases with no known treatments. For drug–drug interaction prediction, heterogeneous graphs capture chemical similarity, shared targets and physiological effects, while subgraph learning and knowledge-enhanced models reveal underlying mechanisms [32,51]. As exemplified by the DDI-GPT framework [32], the integration of KG-derived features with LLMs not only achieves high predictive accuracy but also provides biologically interpretable explanations for the predicted interactions. In drug–target interaction tasks, KGs integrate sequence, structural and semantic information, employing attention-based graph neural networks to capture topological dependencies and uncover actionable drug-target pairs [52,53]. The DTINet framework [52] addresses this by learning low-dimensional representations from a heterogeneous network to predict novel drug-target interactions, with several predictions for cyclooxygenase inhibitors subsequently receiving experimental validation. Beyond interaction prediction, KGs enhance virtual screening, drug recommendation and toxicity assessment by linking patient-specific records, molecular profiles and clinical outcomes, enabling personalized, mechanism-driven decision-making and reducing reliance on costly experimental assays [54,55]. For example, frameworks such as GAMENet [54] integrate DDI knowledge graphs with patient records to recommend safe, personalized medication combinations. Meanwhile, in toxicity assessment, dedicated resources such as ReproTox-KG [55] profile and predict compound-induced birth-defect risks by constructing a specialized knowledge graph. Overall, KGs provide a structured, interpretable and integrative framework that accelerates drug discovery, optimizes therapeutic strategies and improves safety evaluation throughout the drug-development pipeline.

### Omics interpretation and analysis

Omics research, encompassing genomics, transcriptomics, proteomics, metabolomics and microbiomics, underpins systems biology by elucidating molecular architectures of health and disease through multi-scale datasets. The intrinsic complexity and fragmentation of omics data have historically impeded mechanistic insight, but KGs provide a semantically rigorous framework to model entities (e.g. genes, proteins, metabolites, microbes) and their contextual relationships (e.g. regulatory cascades, functional associations, pathophysiological links), enabling cross-disciplinary reasoning, integrative analysis and interpretable hypothesis generation. In genomics, KGs integrate genetic variants, regulatory elements and phenotypic data to move beyond predefined candidate lists towards systems-level inference of gene regulatory mechanisms and pathogenic variants [56–58]. This capability is demonstrated by PhenoKG [58], which leverages graph neural networks to directly infer causative genes from patient phenotypes, providing a powerful framework for rare-disease diagnosis without relying on pre-curated gene panels. Transcriptomic KGs model spatial and regulatory intricacies, capturing intercellular signaling and RNA-mediated regulation through structured representations of ligand-receptor-target pathways and RNA-interaction networks [59,60]. The RNA-KG resource [60] exemplifies this approach, integrating over 60 databases into an ontology-grounded framework that enables systematic exploration of the ‘RNA world’ and its functional implications. Proteomics benefits from graph-based meta-path analyses linking proteins to disease-associated risk genes, facilitating functional annotation, biomarker validation and therapeutic-target prioritization [61,62]. The CKG [62] serves as a prime example of a scalable platform for this purpose, integrating millions of relationships from public databases and the literature to statistically contextualize clinical proteomics data, thereby accelerating the interpretation of biomarker studies and informing clinical decision-making. In metabolomics, KGs contextualize metabolite perturbations within biological networks, uncovering associations with disease phenotypes and supporting biomarker discovery. The FORUM KG [63] addresses the central challenge of biological interpretation in metabolomics by semantically integrating disparate data sources, using ontological reasoning to infer novel metabolite-disease associations and generate testable biological hypotheses from experimental signatures. Microbiomic KGs map ecological and functional interactions between microbial populations, metabolites and host physiology, informing microbe-based therapeutic strategies, as seen in resources such as MMiKG [64] and MiKG[65]. In multi-omics research, KGs unify transcriptomic, proteomic and metabolomic layers into cohesive networks, enabling systems-level modeling of biological processes—for instance, predicting cancer metastasis by integrating graph models with physics-informed constraints [66]. This integrative approach transforms disjointed omics profiles into interpretable, mechanism-driven models that accelerate translational discovery.

### Chemical reaction and synthesis

Chemical reaction mechanism elucidation and compound synthesis are increasingly driven by data- and knowledge-centered approaches. KGs structure chemical entities, reactions, intermediates and properties into graph-based networks, enabling reasoning that accelerates hypothesis generation, predictive modeling and synthesis planning. For reaction prediction, molecules and reactions are represented as nodes and edges, capturing structural similarity, reactivity principles and catalytic dependencies; graph inference and semantic learning facilitate the identification of feasible synthetic routes, prediction of products, intermediates and by-products, and improved reaction classification and yield estimation [67,68]. For instance, the ReaKE framework [68] constructs a chemical synthesis KG to enable contrastive learning that improves reaction classification and product prediction by capturing functional-group transformations. Similarly, CatKG [69] integrates structured reaction databases to model reactant-catalyst-product relations. Through word embeddings and masked language modeling, it supports both analogical reasoning and direct catalyst prediction. In synthesis pathway optimization, KGs integrate data on reactant availability, catalysts and yields, allowing multi-criteria reasoning to identify cost-effective, efficient and sustainable routes. Knowledge-enhanced algorithms and language models are integrated to support dynamic adaptation to new reaction data [8,10,70]. The work by Li *et al.* [70] demonstrates this by building a reaction network KG from historical data to quantify synthetic accessibility, providing a knowledge-based filter for prioritizing synthesizable compounds in molecular design. Moreover, molecular property prediction benefits from situating molecules within networks that link structural features, functional groups and experimental properties, enabling enhanced representation of non-bonding interactions and contextual reasoning for solubility, reactivity and bioactivity, even with limited datasets [5,71,72]. The GODE framework [71] exemplifies this approach by fusing molecular graphs with biochemical KGs through contrastive learning, significantly enhancing property prediction accuracy by leveraging structured domain knowledge. Overall, these KG-based methods provide mechanistic insights and data-driven decision-making in both fundamental and applied chemistry.

### Materials design and discovery

Materials discovery aims to uncover intrinsic links between composition, microstructure and functional properties to accelerate design, performance tuning and scalable application of advanced materials. KGs address the challenge of integrating multi-scale and heterogeneous data by structuring entities and their relationships into semantically rich networks, converting disparate information into actionable knowledge for targeted design, accurate property prediction and efficient screening [73,74]. In new-material design, KGs guide innovative strategies by embedding fundamental principles such as atomic bonding, crystal symmetry and structure-property correlations, enabling the identification of promising candidates in energy, catalysis and nanocomposite materials [75–77], and supporting multi-agent reasoning frameworks for biomimetic materials [31]. For material-performance prediction, KGs integrate elemental, structural and processing information to infer unknown properties through graph traversal and logical reasoning, outperforming conventional simulations in predicting key indicators such as thermal conductivity, mechanical strength, bandgap and formation energy [78,79]. In screening and optimization, KGs consolidate millions of entities across databases, literature and experiments to prioritize candidates with desired characteristics, guide experimental design and balance performance with production feasibility in piezoelectric materials, ultra-high-performance concrete or COFs for gas storage [80–83]. In summary, KGs accelerate materials research by unifying heterogeneous data, enabling predictive performance modeling, and supporting systematic design, screening and optimization of advanced functional materials.

### Summary and prospects

**Figure 6.**
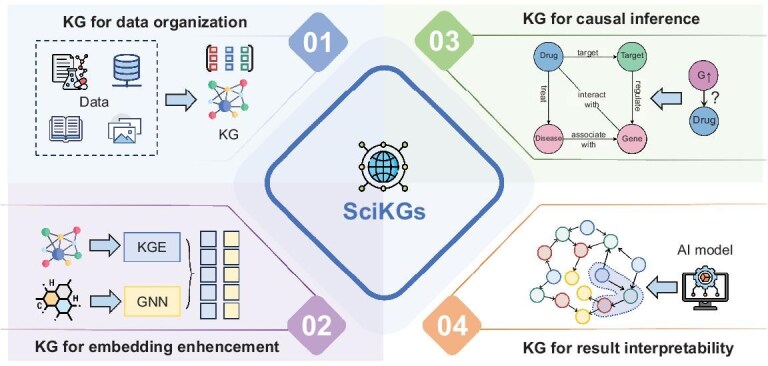
Summary of core functions of SciKGs in diverse scientific tasks. SciKGs serve as a foundational infrastructure that (1) organizes heterogeneous scientific data into structured knowledge; (2) enhances representation learning via graph embedding; (3) enables causal and relational inference for hypothesis generation and (4) improves AI model interpretability by grounding predictions in traceable, evidence-based knowledge paths.

Across the diverse domains of drug discovery, omics analysis, chemical synthesis and materials design, SciKGs have demonstrated their pivotal role as the connective infrastructure of modern scientific intelligence. As summarized in Fig. 6, SciKGs provide a unified framework for knowledge organization, transforming fragmented, multi-source scientific data into coherent, machine-interpretable structures that enable holistic reasoning across molecular, biological and material hierarchies. Through knowledge embedding, SciKGs capture both symbolic semantics and latent correlations, bridging structured ontologies with continuous representations to support predictive and generative modeling. Their graph-based topology further enables causal inference and discovery, allowing automated identification of hidden relationships, such as drug-repurposing candidates that would be difficult to infer from isolated datasets. Beyond predictive performance, SciKGs enhance interpretability and transparency, grounding model outputs in explicit knowledge pathways and causal relationships, thus aligning data-driven predictions with scientific rationale.

While this survey primarily focuses on domains where discrete entities and mature data infrastructures have facilitated early adoption, the underlying graph-structuring paradigm is broadly extensible to the wider AI-for-science landscape. Promising frontiers include physics, where researchers structure physical laws as graph constraints for neural operators, and Earth science, which involves integrating geochemical and atmospheric data to model complex climate feedbacks. These emerging fields introduce distinct challenges, such as handling data sparsity and integrating continuous physical fields with symbolic graphs. Addressing these hurdles will not only broaden the application of SciKGs but also drive the technical evolution of more robust and generalizable knowledge infrastructures.

Looking forward, the next evolution of SciKGs will hinge on cross-disciplinary integration, adaptive intelligence and embodied reasoning. From a systems perspective, future SciKGs should transcend domain boundaries, linking domain-specific knowledge—from atomic interactions to macroscopic physical systems—into interoperable meta-graphs that capture the full continuum. This cross-domain integration addresses critical data-scarcity issues by constructing a unified semantic space. Theoretically underpinned by schema alignment and structural isomorphism, this approach enables hypothesis migration, where logic from data-rich domains (e.g. pharmaceuticals) guides discovery in data-scarce domains (e.g. materials). Recent work such as SciAgents [31] has demonstrated this by leveraging biological mechanisms to inform the generative design of sustainable composites. Furthermore, integrating multi-modal and mechanistic knowledge will foster a new generation of explainable and scientific AI. This convergence of symbolic knowledge, statistical learning and experimental validation points toward a unified paradigm of knowledge-centric scientific discovery, in which SciKGs become both the foundation and the evolving fabric of intelligent, interpretable and autonomous science.

## SYNERGIZING KNOWLEDGE GRAPHS AND LARGE LANGUAGE MODELS

**Figure 7.**
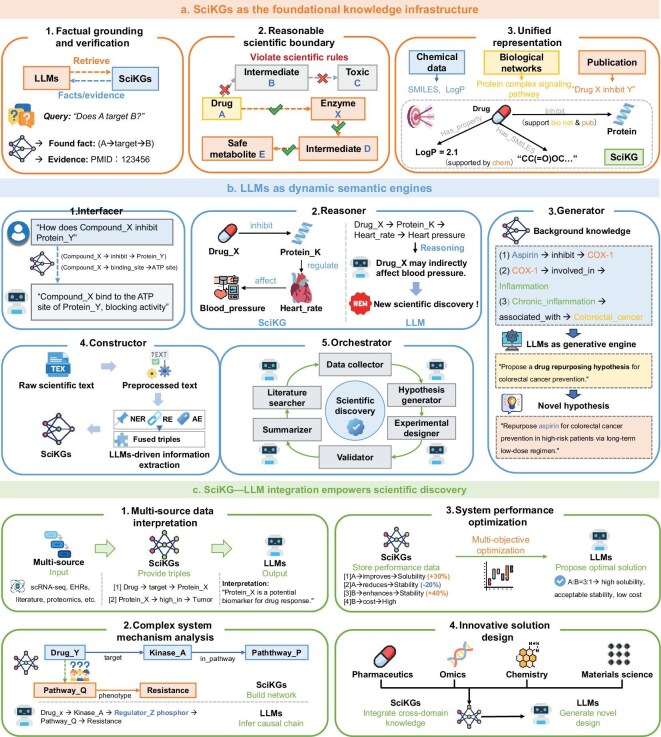
Synergistic integration of SciKGs and LLMs for knowledge-driven scientific discovery. (a) SciKGs serve as the foundational knowledge infrastructure by ensuring factual grounding and verification, defining reasonable scientific boundaries, and enabling unified representation of heterogeneous data. (b) LLMs act as dynamic semantic engines through five core functions: a semantic interface for knowledge access, an analytical reasoner for inference, a generative engine for hypothesis design, a constructor for knowledge curation and an orchestrator for workflow automation. (c) The SciKG–LLM integration empowers four key scientific discovery tasks: multi-source data interpretation, complex-system mechanism analysis, system performance optimization and innovative solution design.

The advancement of scientific discovery increasingly depends on combining knowledge bases with generative intelligence (e.g. LLMs) [31,32,84]. KGs provide explicit representations of entities, relations and domain knowledge, while LLMs offer powerful capabilities in reasoning, abstraction and summary [6,7]. While the integration of KGs and LLMs has been actively explored in general AI contexts, we herein propose a specialized taxonomy tailored for scientific discovery. By synthesizing recent literature, we conceptualize a unified collaborative framework in which SciKGs serve as the foundational knowledge infrastructure and LLMs act as dynamic semantic engines (Fig. 7, Table 1). This synergy enables knowledge-grounded, interpretable and adaptive solutions to complex scientific problems. In this section, we dissect this complementary relationship and its application across the scientific discovery pipeline.

**Table 1.** Representative SciKG–LLM integration systems, categorized by their core functionalities, highlighting the complementary roles of LLMs (as semantic engines) and SciKGs (as knowledge infrastructures) in addressing scientific tasks.

Model	Domains	Roles of LLMs	Roles of SciKGs	Task	Application
KnowNET [86] (2024)	Drug	Semantic interface (query generation)	Grounding (factual verification)	M	Guide health information seeking
FactFinder [87] (2024)	Drug	Semantic interface (query generation)	Grounding (factual retrieval)	M	Life-science question answering
DDI-GPT [32] (2024)	Drug	Reasoner (prediction & explanation)	Representation (semantic enhancement)	C	Explainable prediction of drug-drug interactions
Soman *et al.* [84] (2024)	Drug, omics	Constructor, interface (KG construction, text generation)	Grounding (knowledge base & traceability)	M, C	Drug repurposing and medical QA
BioLORD [99] (2024)	Drug, omics	Reasoner (semantic representation optimization)	Grounding (knowledge base & semantic support)	M	Enhance biomedical semantic similarity
HeCiX [92] (2024)	Drug, omics	Semantic interface (format conversion)	Grounding (knowledge base)	M	Enhance clinical trial research
KRAGEN [95] (2024)	Drug, omics	Orchestrator (plan generation & execution)	Grounding (knowledge base & visualization)	M	Visualized biomedical QA system
MechGPT [96] (2024)	Material	Constructor, reasoner, orchestrator (KG construction, explanation, multi-agent)	Grounding, reasoning constraints (knowledge & explainability)	C, S, I	Materials analysis and design
SciAgents [31] (2024)	Material	Constructor, reasoner, generator (KG construction, analytical reasoning, hypothesis generation)	Grounding (knowledge base)	M, I	Automated discovery in biomaterials science
MKG [51] (2024)	Material	Constructor (KG construction & maintenance)	Grounding (knowledge base)	I	Multidisciplinary materials science discovery
OpenTCM [100] (2025)	Drug	Interface, reasoner, constructor (retrieval, diagnosis, KG construction)	Reasoning constraints (knowledge retrieval enhancement)	M	Traditional Chinese medicine diagnosis
iKraph [9] (2025)	Drug	Constructor (KG construction)	Grounding (knowledge base)	S	Biomedical research
KGT [15] (2025)	Drug, omics	Interface, reasoner (query generation & reasoning output)	Grounding, reasoning constraints (fact checking & path constraint)	S, M	Drug repositioning, framework for pan-cancer QA
ESCARGOT [94] (2025)	Drug, omics	Generator, orchestrator (strategy & code generation)	Grounding (knowledge base)	S, I	Biomedical AI agent
Cat-KG [90] (2025)	Chemistry	Constructor, reasoning, interface (KG construction, path reasoning & explanation)	Grounding, reasoning constraints (explainability & path constraint)	C, M	Relay catalysis pathway recommendation
Ma *et al.* [10] (2025)	Chemistry	Constructor, generator (KG construction & path recommendation)	Grounding (structured knowledge management)	S	Automated Retrosynthesis Planning of macromolecules
KG-FM [82] (2025)	Material	Constructor, reasoner (multi-modal extraction, QA & reasoning)	Grounding (knowledge base & visualization)	M	Improve LLM QA in framework materials
SciToolAgent [97] (2025)	Comprehensive	Orchestrator (multi-agent collaboration)	Grounding (tool knowledge base)	S, M, I	Scientific agent for multi-tool integration

M, multi-source data interpretation; C, complex-system mechanism analysis; S, system performance optimization; I, innovative solution design.

### SciKGs as the foundational knowledge infrastructure

Traditional LLMs are prone to hallucinations during scientific reasoning, such as generating non-existent drug-target interactions, which leads to outputs lacking factual support [85]. Moreover, LLMs struggle with grounding in physical constraints, reliable generation of symbolic structures and robustness to domain shifts—limitations that are particularly concerning in safety-critical applications such as drug discovery. Leveraging their explicit entity-relationship structure, SciKGs constrain LLM reasoning from three key perspectives to ensure the reliability of scientific decision-making. First, SciKGs ensure factual grounding, verification and precise retrieval. They serve as authoritative benchmarks against which LLM-generated hypotheses can be validated, directly countering the hallucinations and factual inconsistencies. For instance, the KNOWNET framework [86] extracts triples from LLM outputs and maps them to validated evidence in external KGs, providing a visual interface to trace and verify claims. Similarly, FactFinder [87] augments LLMs with a medical KG through a structured retrieval-and-generation pipeline, which retrieves precise subgraphs to focus LLM attention and demonstrates significant improvements in the accuracy and completeness of responses for critical tasks such as target identification. The efficacy of these systems, however, is inherently tied to the completeness and veracity of the KG itself; missing or erroneous facts in the KG can lead to false validation or missed detections. By accessing pre-stored knowledge of established scientific mechanisms, these systems assess the plausibility of proposed ideas and provide traceable evidence, directly countering the opaque nature of black-box LLM reasoning [88]. Second, SciKGs define reasonable boundaries for explainable causal reasoning. They prevent the generation of schemes that violate scientific principles, thus addressing the LLM’s lack of intrinsic grounding in physical laws and domain constraints. The graph-constrained reasoning (GCR) framework [89] integrates the KG structure directly into the LLM’s decoding process, ensuring that every step of the reasoning path remains faithful to the graph and preventing attention drift, thereby achieving zero reasoning hallucination in knowledge-graph question-answering tasks. In chemistry, the synergizing KG and LLM approach [90] for relay catalysis uses a detailed catalysis KG (Cat-KG) to apply expertise-informed scoring rules, ensuring that only chemically plausible multi-step reaction pathways are recommended and thereby constraining the LLM’s generative space to scientifically valid outcomes. Finally, advanced multi-modal SciKGs integrate heterogeneous data into a unified framework, allowing LLMs to perform cross-modal reasoning and holistic analyses while mitigating their brittleness to domain shifts. Systems such as DDI-GPT [32] exemplify this by constructing a multi-modal KG that fuses drug-related chemical, substructure and molecular data. This rich, structured context enables the LLM not only to predict drug-drug interactions with high accuracy but also to generate explainable insights grounded in multifaceted evidence from the graph, providing a scaffold for more reliable symbolic and structured reasoning. Beyond these functional capabilities, SciKGs offer a fundamental advantage in dynamic knowledge evolution. Unlike foundation models, which require computationally expensive retraining to absorb new findings, SciKGs support incremental, low-cost updates, enabling the real-time integration of emerging discoveries. In the era of powerful foundation models, SciKGs are therefore not optional but essential infrastructure. They act as the irreplaceable deterministic substrate that complements the probabilistic nature of LLMs and provide the explicit provenance, symbolic logic, multi-modal consistency and dynamic evolvability required for rigorous AI-driven science.

### LLMs as dynamic semantic engines

Despite their strengths in structured representation, SciKGs are inherently static, presenting a fundamental challenge for dynamic scientific exploration [91]. LLMs bridge this gap by serving as dynamic semantic engines that transform static knowledge into actionable scientific intelligence. This transformation is achieved through several core capabilities. First, LLMs act as semantic interfaces, parsing complex SciKGs and converting structured scientific data into intuitive natural language summaries and precise formal queries. Systems such as HeCiX [92] demonstrate this by integrating a biomedical KG with GPT-4 through LangChain, creating a natural language interface that enables researchers to efficiently query complex clinical-trial and biological data. This dramatically lowers the barrier to cross-domain knowledge acquisition and facilitates interdisciplinary collaboration. Second, they function as analytical reasoners, performing complex inference and prediction tasks based on the rich relational structures of SciKGs to uncover novel mechanistic insights. The DDI-GPT framework [32] exemplifies this capability, where an LLM enhanced with a multi-modal drug KG not only predicts drug-drug interactions with high accuracy but also generates explainable insights by capturing contextual dependencies between biomedical entities. Third, they serve as generative engines for scientific innovation, producing novel and plausible hypotheses, experimental strategies and design solutions that are grounded in structured knowledge. SciAgents [31] showcases this through a multi-agent system that autonomously generates and refines research hypotheses for bioinspired materials discovery by leveraging large-scale ontological knowledge graphs. Similarly, the automated retrosynthesis planning system by Ma *et al.* [10] demonstrated how LLMs can design novel synthesis pathways for macromolecules by extracting and reasoning over chemical reaction data stored in KGs. Furthermore, LLMs undertake a constructive role by building, curating and maintaining SciKGs from raw scientific literature and data. The comprehensive biomedical KG iKraph [9] exemplifies this, where an LLM-powered information-extraction pipeline processes all PubMed abstracts to construct a large-scale KG that matches human expert annotations. The KG-RAG framework [84] further demonstrates how LLMs can optimize knowledge extraction from biomedical KGs in a token-efficient manner, while systems such as UpToDate [93] show how LLMs can automatically validate and update KG facts to maintain currency. Finally, in their most advanced role, LLMs orchestrate complex scientific workflows, managing multi-step reasoning processes and coordinating multi-agent systems. The ESCARGOT agent [94] exemplifies this by combining LLMs with a dynamic Graph of Thoughts and biomedical KGs to significantly outperform standard retrieval-augmented-generation methods in open-ended biomedical questions. These functions position LLMs as active collaborators in scientific discovery, transcending mere information retrieval.

### SciKG–LLM integration empowers scientific tasks

Built on the complementary roles of factual anchors and semantic engines, the SciKG–LLM synergy framework can systematically address four core tasks in scientific discovery, covering the full workflow from fundamental cognition to applied breakthroughs (Table 1). During multi-source data interpretation, SciKGs convert massive datasets into structured triples, and LLMs extract interpretable knowledge to unlock the latent value of data accumulation [51,94]. For complex-system mechanism analysis, SciKGs integrate multi-source data to construct entity-relationship networks, and LLMs infer causal chains based on these networks, addressing the limitation of traditional methods that prioritize phenomenological description over causal modeling [86,87,95]. In system-performance optimization, SciKGs store quantitative variable–performance correlations, and LLMs generate multi-objective optimal solutions by incorporating domain constraints to overcome the local optimization trap of trial-and-error iteration [32,84,90]. For innovative scheme design, SciKGs integrate cross-domain knowledge, and LLMs generate new schemes that combine multidisciplinary principles through analogical reasoning, helping to overcome the innovation lag caused by domain barriers [9,10]. Overall, these four tasks constitute a self-reinforcing discovery feedback loop. The process begins with multi-source data interpretation, which feeds structured knowledge into the SciKG. This enriched graph enables deeper complex-system mechanism analysis, whose insights guide system-performance optimization. The optimized systems and improved mechanistic understanding then fuel innovative solution design, which, when experimentally validated, generates new data and knowledge, thus closing the loop and beginning the cycle anew.

### Toward the autonomous scientific discovery paradigm

**Figure 8.**
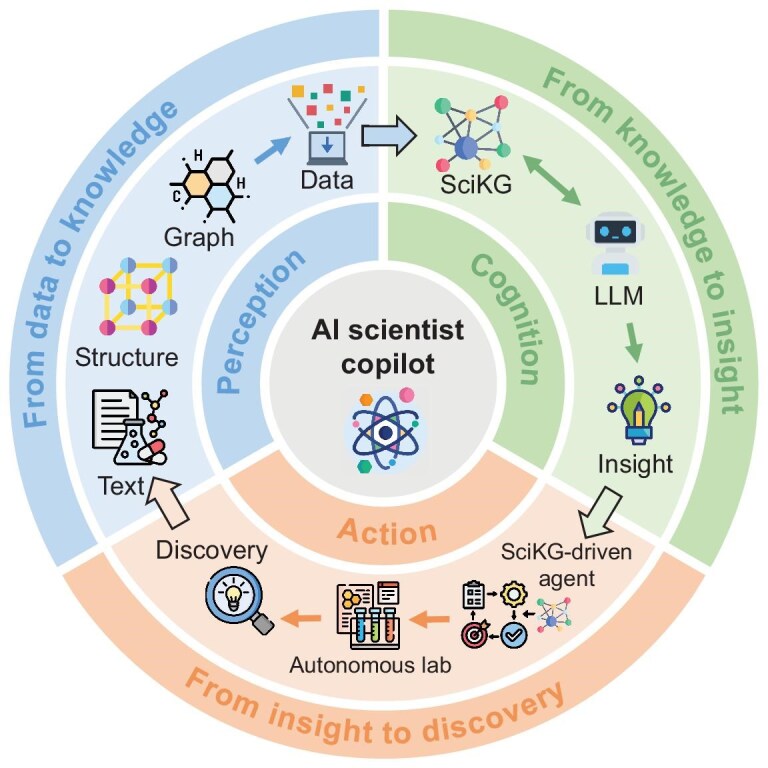
The autonomous scientific discovery flywheel driven by LLM agents and SciKGs.

The synergistic integration of SciKGs and LLMs heralds a paradigm shift in scientific methodology, from human-driven hypothesis–validation cycles to AI-augmented autonomous discovery loops. In this emerging paradigm, LLMs continuously generate and refine hypotheses from massive multi-modal data; SciKGs evaluate and ground these hypotheses against existing knowledge; and validated results are automatically integrated back into the SciKG, forming an ever-growing knowledge flywheel. This closed feedback loop enables a self-evolving scientific ecosystem capable of accelerating discovery at scale. The concrete embodiment of this framework is the *AI Scientist Copilot*: an autonomous system that embeds the SciKG-LLM synergy within a perception-cognition-action loop to assist and augment the scientific process (Fig. 8). Such a copilot is capable of sensing, reasoning and acting across the full discovery pipeline.

1. *From data to knowledge.* LLMs act as perceptual organs that ‘understand’ scientific literature and multi-modal data, while SciKGs provide the structured schema to organize this extracted information. This synergy transforms raw data into computable knowledge across domains. For instance, in biomedicine, iKraph [9] employs LLMs to extract entities and relations from PubMed abstracts, constructing a comprehensive biomedical KG comparable to human curation. Similarly, in chemistry, LLMs parse reaction literature to populate KGs with precise synthesis conditions [70], while in materials science, frameworks such as MatKG [74] utilize LLMs to integrate heterogeneous data from publications and simulations into unified graphs. In these systems, LLMs unlock the information, and SciKGs store it in a rigorous, machine-interpretable format.
2. *From knowledge to insight.* SciKGs function as long-term memory and logical engines, while LLMs perform analogical reasoning and path inference. Their collaboration enables verifiable reasoning chains: LLMs hypothesize causal links, and SciKGs validate or refine these paths with literature evidence or alternative mechanisms. Such collaborative reasoning drives insights across domains: it generates biologically grounded explanations for drug-drug interactions (e.g. DDI-GPT [32]), identifies chemically plausible reaction pathways in catalysis (e.g. Cat-KG [90]) and contextualizes property predictions for new materials (e.g. MechGPT [96]). This bidirectional flow, in which LLMs propose and SciKGs ground, ensures that generated insights are both innovative and scientifically sound.
3. *From insight to discovery.* LLMs operate as strategy planners, designing experiments, synthesis routes or material compositions; SciKGs act as feasibility filters, ensuring that proposed actions comply with known scientific principles. This capability is exemplified by systems such as SciAgents [31], where a multi-agent framework reasons over a materials knowledge graph for bioinspired design; Automated Retrosynthesis Planning [10], which uses reaction KGs to constrain and validate LLM-generated synthesis pathways; and SciToolAgent [97], which leverages a scientific tool knowledge graph to automatically orchestrate the execution of complex analytical workflows (e.g. protein design, chemical reactivity prediction and MOF materials screening). The physical realization of this loop is now being demonstrated by autonomous robotic platforms, such as multi-agent driven robotic AI chemists that conduct closed-loop chemical research on demand [98]. Coupled with automated laboratory systems, this architecture can close the loop from computational inference to physical experimentation.

### Summary and prospects

In summary, SciKG–LLM synergy marks a conceptual leap from knowledge utilization to knowledge evolution. By combining SciKGs with the generative and reasoning power of LLMs, future scientific ecosystems may operate as continuously learning systems, capable of generating, testing and consolidating knowledge without constant human supervision.

Realizing this vision, however, requires a clear-eyed assessment of the paradigm’s inherent fragility. The reliability of SciKG-LLM systems is co-dependent: the veracity of the SciKG (subject to incompleteness, noise and ontological misalignment) directly grounds or misguides LLM reasoning; conversely, LLM imperfections (hallucinations, biases and over-generalization) can propagate errors during knowledge extraction, curation and generative design, potentially corrupting the very knowledge infrastructure on which they rely. This bidirectional risk is amplified in autonomous discovery loops, where errors may compound. Developing robust evaluation protocols to measure factuality, reasoning robustness and performance gains, summarized in Table S9, is therefore a critical step toward trustworthy autonomous discovery.

These inherent vulnerabilities become particularly acute as systems evolve from passive retrieval toward active, autonomous generation. As these systems evolve toward autonomy, a critical frontier lies in developing guardrails for generative science. Existing works have yet to fully resolve how to prevent plausible but scientifically invalid hallucinations in experimental design. Future research must focus on establishing a tiered constraint framework: (i) *feasibility checks*, where SciKGs act as physical logic filters (e.g. verifying reagent compatibility); (ii) *safety and ethical compliance*, integrating toxicity and biosafety protocols directly into graph attributes; and (iii) *scientist-in-the-loop governance*, ensuring that high-stakes autonomous decisions trigger expert-review checkpoints. Addressing these validation challenges is a prerequisite for deploying trustworthy AI scientist copilots.

The next frontier lies in building robust, safety-aware LLM-based scientist copilots that embody this closed-loop intelligence, integrating real-time experimental feedback with cognitive models to achieve a new era of autonomous scientific discovery.

## LIMITATIONS, CHALLENGES, OPPORTUNITIES AND FUTURE DIRECTIONS

**Figure 9.**
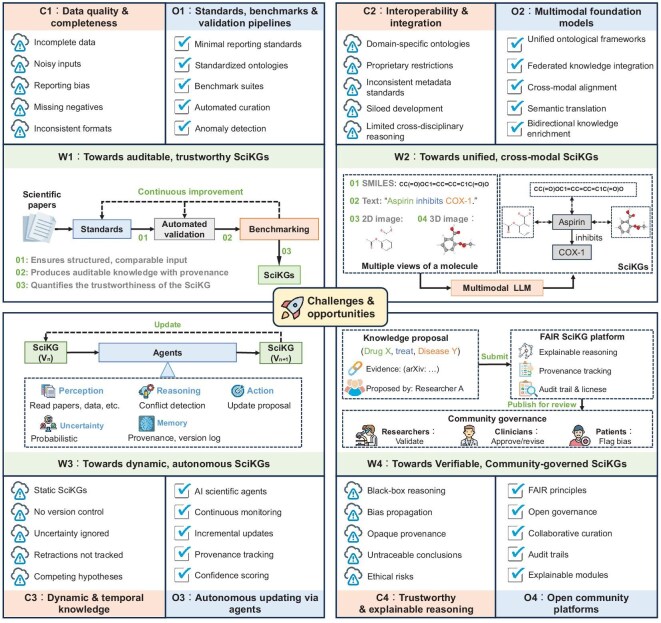
Challenges and opportunities of SciKGs. This figure illustrates the major challenges (C1–C4) facing SciKGs, including data quality and completeness, interoperability and integration, dynamic and temporal knowledge, and trustworthy and explainable reasoning. Each challenge is paired with corresponding opportunities (O1–O4) for advancement, such as building standards and benchmarks, integrating multi-modal foundation models, autonomous updating via agents and developing community-driven platforms. The sections depicting workflows (W1–W4) that enable these opportunities, highlighting a path towards more auditable, unified, dynamic and community-governed SciKGs.

Despite the growing success, SciKGs remain in an early stage of development. Their evolution is shaped not only by technical challenges but also by fundamental epistemological constraints and practical barriers. In this section, we first discuss the inherent limitations of the SciKG paradigm itself. We then examine four major technical challenges—data quality, interoperability, dynamism and trustworthy reasoning—and highlight promising opportunities for advancing SciKGs (Fig. 9). We further propose three complementary directions for next-generation SciKGs, aimed at enhancing their role as actionable knowledge infrastructures for scientific discovery.

### Inherent limitations of SciKGs

#### Oversimplification of continuous processes

The discrete triple structure (subject–predicate–object) inherent to KGs often fails to capture the continuous, dynamic nature of scientific processes. For example, a simple ‘drug–inhibits–protein’ relation may overlook critical contextual factors such as dosage dependence, temporal dynamics, spatial localization or reaction kinetics. This structural limitation can lead to a loss of mechanistic detail, restricting the graph’s ability to fully represent complex biological, chemical or physical phenomena.

#### Epistemic uncertainty and retraction handling

Scientific knowledge is continually revised as new evidence emerges, yet most SciKGs treat extracted relations as static facts. Current frameworks lack built-in mechanisms to represent confidence scores, conflicting hypotheses or negative results. Moreover, when published findings are retracted or corrected, propagating those updates through the graph while ensuring downstream models disregard invalidated triples remains an unresolved challenge. This can lead to persistent error propagation and reduce the reliability of KG-driven predictions.

#### Domain imbalance and bias

SciKGs constructed from the literature inevitably inherit the severe research biases present in scientific publishing. Well-studied entities (e.g. certain disease-associated genes or high-performance materials) accumulate dense connections, while under-researched areas remain sparse. This ‘rich-get-richer’ topology can skew graph algorithms, such as embedding methods or link-prediction models, toward highly connected nodes, potentially overlooking novel interactions or under-representing emerging scientific domains.

### Technical challenges

#### Data quality and completeness

The effectiveness of SciKGs depends critically on the quality, consistency and coverage of the underlying data. Scientific data are often incomplete, noisy or biased, reflecting variations in experimental protocols, reporting standards and publication practices. For example, biomedical databases may lack negative results, while materials datasets may disproportionately emphasize high-performing compounds. Integrating heterogeneous sources further compounds these issues, as differences in terminology, granularity and measurement standards lead to inconsistencies that propagate through the graph. Ensuring robust data quality requires advances in automated curation, normalization and error detection, as well as community-wide efforts to establish minimal reporting standards and promote the publication of negative and null results. Ultimately, improving data quality and completeness will determine whether SciKGs can provide trustworthy substrates for scientific reasoning.

#### Interoperability and integration

A second challenge lies in the integration of knowledge across diverse scientific disciplines. Existing SciKGs are often domain specific, relying on bespoke ontologies that impede interoperability across biology, chemistry and materials science. Beyond bespoke schemas, integration faces dual hurdles: technical heterogeneity and practical access barriers. Technically, scientific concepts often suffer from cross-disciplinary polysemy (e.g. ‘nucleus’ in cell biology versus physics) and topological disparities (e.g. dense interaction networks versus sparse property tables), complicating schema alignment. Practically, significant barriers arise from restrictive data governance. Many high-value datasets are locked behind proprietary paywalls or restrictive licenses, creating ‘data silos’ that effectively block the construction of comprehensive public graphs. Even when data are accessible, inconsistent usage policies further complicate automated federation. This siloed development undermines the potential of SciKGs to support cross-disciplinary reasoning, for instance, linking protein interaction networks with materials-based drug delivery systems. Addressing these multifaceted challenges requires a synergistic approach: deploying technical solutions such as automated ontology alignment and federated query architectures to resolve heterogeneity, while simultaneously advocating for standardized open-data licensing to break down proprietary silos. Such advances would enable truly cross-domain SciKGs that capture the interconnected nature of scientific discovery.

#### Dynamic and temporal knowledge

Scientific knowledge is inherently dynamic, with new discoveries, revised hypotheses and retracted claims constantly reshaping the research landscape. Traditional KGs, however, are largely static, making them ill suited to capture the temporal and evolving nature of science. This mismatch raises both technical and epistemic challenges: how can SciKGs be continuously updated without sacrificing reproducibility? How should they represent uncertainty, competing hypotheses or retracted findings? Current frameworks lack mechanisms to encode confidence scores, conflicting evidence or the provenance of retracted claims. Probabilistic knowledge graphs (modeling triples with associated probabilities) offer a promising direction for representing uncertainty, but their scalable integration and efficient reasoning remain open problems. Incremental learning and temporal graph modeling offer promising solutions, enabling knowledge graphs to evolve in tandem with scientific progress. At the same time, reproducibility concerns highlight the need for version-controlled and provenance-aware SciKGs, ensuring that dynamic updates remain transparent and traceable.

#### Trustworthy and explainable reasoning

As SciKGs are increasingly coupled with LLMs, questions of trust, transparency and bias become paramount. Automated reasoning over incomplete or biased data risks producing misleading conclusions, with potentially serious implications in sensitive domains such as drug development or clinical decision-making. The lack of formal uncertainty representation (e.g. confidence scores for triples) and mechanisms to handle retractions or conflicting findings undermines the trustworthiness of KG-driven inferences. Moreover, the opaque nature of many AI models undermines interpretability and hinders adoption by domain experts. Building trustworthy SciKGs requires mechanisms for explainable reasoning, bias detection and mitigation, and transparent provenance tracking. Ethical and societal considerations must also be addressed, including issues of data privacy, intellectual property and equitable access to knowledge infrastructures. Establishing trustworthiness is not merely a technical challenge but a prerequisite for integrating SciKGs into the scientific process.

### Opportunities

#### Building standards, benchmarks and validation pipelines

To address data quality at scale, there is a pressing need for community-driven standards, benchmarking frameworks and automated validation pipelines. Establishing minimal information standards across domains ensures consistent and transparent reporting. Standardized ontologies and interoperable data formats enable harmonization across repositories, while benchmark suites can evaluate SciKG performance in capturing domain-specific knowledge, such as chemical reaction mechanisms in chemistry or gene regulatory networks in biology. Furthermore, automated curation tools powered by natural language processing and machine learning can detect anomalies, impute missing values and flag potential biases. Together, these measures create a feedback loop of continuous quality assessment and improvement, transforming SciKGs into auditable and trustworthy knowledge infrastructures.

#### Deeper integration with multi-modal foundation models

To bridge disciplinary divides, SciKGs must evolve into multi-modal, semantically unified knowledge backbones that integrate diverse data types and modalities. Foundational multi-modal LLMs can act as powerful intermediaries for cross-modal alignment and semantic translation. For example, LLMs can extract and normalize entity mentions from scientific texts across domains, while molecular encoders standardize chemical representations. When grounded in a shared SciKG schema, these models enable knowledge fusion across text, tables, images and structured databases. This bidirectional integration allows foundation models to enrich SciKGs with newly mined knowledge, while SciKGs provide symbolic, interpretable constraints that improve the factual accuracy and reasoning fidelity of generative models. The result is a synergistic architecture that supports truly interdisciplinary knowledge synthesis.

#### Autonomous updating and correcting knowledge graphs via LLM agents

To keep pace with the evolving nature of science, SciKGs must transition from static repositories to adaptive, self-updating systems. Autonomous scientific agents capable of reading literature, analyzing data, generating hypotheses and even designing experiments can serve as intelligent curators that continuously monitor, update and validate knowledge graphs. These agents can perform incremental updates, flag anomalies, resolve contradictions using probabilistic reasoning and maintain versioned histories of assertions with full provenance. They can also attach confidence scores to triples based on evidence strength and implement procedures for deprecating knowledge linked to retracted publications. For instance, in genomics, an agent could detect conflicting annotations about a gene’s function, assess the credibility of sources and dynamically update the graph with confidence scores. Similarly, in materials science, agents could ingest newly published alloy properties and suggest plausible performance predictions. By embedding temporal logic and uncertainty modeling, such agent systems transform SciKGs into evolving knowledge ecosystems.

#### Developing open SciKG platforms

To establish trust, SciKGs must be developed and governed through open, inclusive and community-led platforms grounded in the FAIR principles: findability, accessibility, interoperability and reusability. Such platforms empower diverse stakeholders to collaboratively build, validate and govern knowledge graphs. Crucially, to dismantle the practical barriers created by proprietary restrictions (as highlighted in the Challenges section), the community must adopt transparent open data licenses (e.g. CC-BY) and develop sustainable business models for maintaining public knowledge infrastructures. Transparent provenance tracking, open licensing and audit trails ensure accountability, while modular, explainable reasoning modules allow users to trace how conclusions are derived. For example, global biomedical consortia could co-develop a shared SciKG integrating clinical trial data, omics profiles and real-world patient outcomes, enabling transparent and reproducible translational research. By democratizing access and participation, these platforms not only enhance trustworthiness but also foster equitable innovation across regions and disciplines.

### Future directions

#### SciKG self-evolving framework

Future SciKGs should be designed as self-evolving frameworks capable of autonomously ingesting new knowledge, detecting inconsistencies and refining existing entities, relations and attributes. Realizing such self-evolution can be conceptualized as a multi-agent system, in which specialized agents handle complementary tasks: one agent continuously mines and extracts new knowledge from publications, preprints or experimental logs; another agent performs consistency checking, conflict resolution and uncertainty quantification; yet another updates embeddings and temporal representations while maintaining provenance and version control. These agents communicate and coordinate to ensure that the knowledge graph evolves in a coherent and reproducible manner. Methodologically, incremental learning, temporal graph modeling and probabilistic graph models underpin agent operations, while automated pipelines powered by LLMs enable the ingestion of unstructured text and multi-modal data. For example, in genomics, an extraction agent could identify new functional annotations for genes, a validation agent could reconcile conflicts with existing evidence and an update agent could adjust confidence scores for prior assertions. By framing self-evolution as a coordinated multi-agent system, SciKGs achieve adaptive knowledge management, supporting longitudinal studies and scalable, automated curation across scientific domains.

#### SciKG–LLM co-evolution system

A co-evolutionary framework between SciKGs and LLMs envisions a tightly coupled system in which structured and unstructured knowledge continuously inform and refine one another through an iterative, bidirectional pipeline. In this paradigm, LLMs equipped with domain-specific prompting, retrieval-augmented generation and self-verification modules autonomously extract new entities, relations and hypotheses from scientific literature, experimental logs and multi-modal datasets. The extracted triples are then verified by a knowledge validation agent that applies probabilistic reasoning and schema alignment to ensure consistency and novelty before being merged into the SciKG via incremental updates with full provenance tracking. Conversely, SciKGs serve as interpretable priors that ground and constrain LLM inference, reducing hallucinations and enhancing domain fidelity through techniques such as graph-based retrieval augmentation, neural-symbolic reasoning and contrastive knowledge alignment that integrate KG embeddings directly into the LLM’s representation space. Over time, co-adaptive feedback mechanisms allow both components to improve jointly: the evolving SciKG provides structured supervision for continual fine-tuning or reinforcement learning of the LLM, while the LLM reorganizes and corrects graph regions showing inconsistency or conceptual drift. This closed feedback loop enables SciKGs to grow richer and more precise while LLMs become more grounded and interpretable, forming a foundation for more reliable and explainable scientific reasoning.

#### SciKG-driven AI scientist agents

The ultimate vision is to embed SciKGs within autonomous AI scientist agents that operate in a closed-loop ‘perception–cognition–execution–feedback’ cycle. In this paradigm, agents perceive experimental or computational data, encode it into the SciKG, cognitively reason over the integrated knowledge (using both symbolic reasoning and generative LLM inference) and plan subsequent actions, including designing new experiments or simulations. Key components include reinforcement learning for action selection, thinking and reasoning frameworks to handle uncertainty and conflicting evidence, and automated experiment-execution interfaces (e.g. robotic laboratory platforms). For instance, in materials discovery, the agent could propose a new alloy composition, simulate its thermodynamic stability, update the SciKG with predicted properties and decide the next set of experiments based on expected information gain. The closed-loop integration of real-time data ingestion, knowledge graph updates and adaptive action planning enables a continuously learning system, in which the SciKG serves not only as a repository of knowledge but also as a dynamic decision-making substrate that informs, constrains and amplifies scientific exploration.

In summary, these directions envision SciKGs not merely as static repositories but as the dynamic reasoning core of future scientific ecosystems. The progression from self-evolving frameworks to co-evolution with LLMs, and ultimately to embodiment within AI scientist agents, charts a course toward autonomous discovery systems. By pursuing this roadmap, SciKGs can evolve from passive knowledge bases into active partners in the scientific process, capable of guiding, accelerating and ultimately redefining the very frontiers of scientific exploration.

## Supplementary Material

nwag140_Supplemental_File
