# Supplementary material for: Bridging data and discovery: a survey on knowledge graphs in AI for science
Source: Natl Sci Rev. 2026 Mar 5;13(8):nwag140. doi: 10.1093/nsr/nwag140 (PMC13154823; doi:10.1093/nsr/nwag140)
Supplement: nwag140_Supplemental_File [file nwag140_supplemental_file.pdf]

Supplementary Information for
"Bridging Data and Discovery: A Survey on Knowledge Graphs in AI for Science"

Keyan Ding[1,2,†], Zhihui Zhu[2,†], Yuqi Tang[3], Kehua Feng[1], Xiang Zhuang[1,4], Hongwei Wang[3], Yi Yang[1], Huifang Du[5], Zhangkai Ni[6], Shiqi Wang[7], Xiaohui Fan[8], Huabin Xing[9], Lei Bai[4,*], Qi Liu[10,*], Haofen Wang[5,*], Qiang Zhang[2,3,*], and Huajun Chen[1,2,*]

[1]College of Computer Science and Technology, Zhejiang University, Hangzhou 310027, China

[2]ZJU-Hangzhou Global Scientific and Technological Innovation Center, Zhejiang University, Hangzhou 311200, China

[3] ZJU-UIUC Institute, Zhejiang University, Haining 314400, China

[4] Shanghai Artificial Intelligence Laboratory, Shanghai 200232, China

[5] College of Design and Innovation, Tongji University, Shanghai 200092, China

[6] College of Computer Science and Technology, Tongji University, Shanghai 201804, China

[7] Department of Computer Science, City University of Hong Kong, Hong Kong 999077, China

[8] College of Pharmaceutical Sciences, Zhejiang University, Hangzhou 310058, China

[9] College of Chemical and Biological Engineering, Zhejiang University, Hangzhou 310027, China

[10] School of Life Sciences and Technology, Tongji University, Shanghai 200092, China

† **Equal contribution.**
* **Corresponding authors:** baisanshi@gmail.com, qiliu@tongji.edu.cn; haofen.wang@tongji.edu.cn; qiang.zhang.cs@zju.edu.cn; huajunsir@zju.edu.cn.

List of Supplementary Tables

S1 Commonly Used Databases for Scientific Knowledge Graph Construction 3
S2 Commonly Used Software and Tools for Scientific Knowledge Graph Construction and Management 4
S3 Comparative analysis of knowledge extraction paradigms in scientific domains 5
S4 Taxonomy of evaluation methodologies for Scientific Knowledge Graphs 6
S5 Representative SciKGs for Drug Development and Optimization 7
S6 Representative SciKGs for Omics Interpretation and Analysis 8
S7 Representative SciKGs for Chemical Reaction and Synthesis 9
S8 Representative SciKGs for Materials Design and Discovery 10
S9 Evaluation practices for KG-LLM integration in scientific discovery. 11

Table S1: Commonly Used Databases for Scientific Knowledge Graph Construction

Domain	Database	Short description	Statistics	Update Frequency
Drug Databases	BindingDB [1]	Publicly accessible collection of measured drug-target binding affinities.	3.1M binding data for 1.3M compounds & 9.6K targets.	Weekly
	DrugBank [2]	Richly annotated resource combining drug data with target, pathway & pharmacogenomic info.	18K approved & investigational drugs, 23K drug-target links, 3.6K drug-transporter links, 6K drug-enzyme links.	Monthly
	CTD [3]	The comparative toxicogenomics database links chemicals, genes, phenotypes and diseases.	101M toxicogenomic interactions, 19K chemicals, 57K genes, 7K diseases.	–
	DisGeNET [4]	Comprehensive platform integrating genes, variants, and human diseases, combining curated data and text-mined evidence.	2.0M gene–disease associations, 4.4M variant–disease associations, and 28M disease–disease associations	–
	DrugCentral [5]	Authoritative, open-access compendium of active pharmaceutical ingredients approved worldwide.	5K drugs, 152K pharmaceutical products.	–
	PharmGKB [6]	Provide PGx data from literature annotations to genotype-based treatment guidelines.	209 clinical guideline annotations, 1.2K drug label annotations, 483 FDA drug label annotations	–
	SIDER [7]	Database of marketed drugs and their recorded adverse drug reactions (ADRs).	1.4K drugs, 6K side effects, 140K drug–side effect pairs.	Static
Omics Databases	Uniprot [8]	Comprehensive, high-quality protein sequence & functional annotation database.	573K reviewed entries, 253K unreviewed entries.	4 Weeks
	Ensembl [9]	Genome browser & annotation resource for vertebrates and selected eukaryotes.	300+ species, 40K coding genes (human), 1M variants.	3 Months
	KEGG [10]	Database integrating pathways, genes, compounds, drugs and diseases for system analysis.	75K pathways, 54 M genes, 12K drugs, 11K diseases.	Daily
	Reactome [11]	Curated, peer-reviewed pathway database emphasizing human biology.	2.8K human pathways covering 11.6K proteins, 16K reactions.	Monthly
	InterPro [12]	Comprehensive resource integrating multiple protein signature databases.	13 member databases covering millions of protein sequences.	Quarterly
	RNAcentral [13]	Comprehensive ncRNA sequence collection representing all ncRNA types across diverse organisms.	44.5M non-coding RNA sequences, covering 1.1K species from 54 databases.	Twice a year
	STRING [14]	Database of known and predicted protein–protein interactions across multiple organisms.	59.3M proteins, 20B PPIs, 12.5K organisms	–
	MONDO† [15]	Ontology harmonizing disease concepts with standardized identifiers, mappings, and classifications for clinical use.	17 disease resources integrated into 22K unified disease concepts.	Monthly
	UMLS [16]	Comprehensive biomedical ontology integrating multiple vocabularies to unify concepts, names, and relationships.	17M names, 3.4M concepts, 8.7M codes, 190 vocabularies, 29 languages	Twice a year
Chemical Database	ChEBI [17]	Chemical entities of biological interest, a dictionary and ontology of small molecular entities.	62K compounds.	Monthly
	ChEMBL [18]	A curated database of drug-like bioactive molecules that integrates chemical, bioactivity and genomic data to support drug discovery.	2.5M compounds, 1.7M assays, 15.5K drugs, 48.8K drug indications.	–
	Reaxys [19]	Elsevier-curated chemical reactions, substances, properties & literature.	283M chemical substances, 73M reactions, 500M physicochemical data points.	–
	PubChem [20]	NIH repository of chemical substances, bioactivities & patents.	122M compounds, 338M substances, 297 M bioactivities.	Daily
	ZINC [21]	Free database of commercially available compounds for virtual screening.	980M purchasable compounds.	–
Materials Databases	OQMD [22]	Open-access database of DFT-calculated properties for inorganic and hybrid materials.	1.2M materials.	–
	Materials Project [23]	High-throughput DFT database of materials properties & crystal structures.	144K inorganic compounds, 76K bandstructures, 64K molecules, 530K nanoporous materials, and diverse tensors and electrodes.	–

Table S2: Commonly Used Software and Tools for Scientific Knowledge Graph Construction and Management

Category	Software Name	Short Description	Supported Tasks	License
Automated KG Construction	DeepKE[24]	A knowledge extraction toolkit for knowledge graph construction supporting cnSchema, low-resource, document-level and multimodal scenarios.	Named Entity Recognition, Relation Extraction, Attribute Extraction, etc.	MIT License
	OneKE[25]	A flexible dockerized system for schema-guided knowledge extraction, capable of extracting information from web and PDF books.	Named Entity Recognition, Web News Extraction, Book Knowledge Extraction, etc.	MIT License
	AutoKG[26]	An LLM-powered multi-agent framework for automated KG construction and reasoning, integrating external knowledge sources.	Entity/Relation Extraction, KG Construction, KG Reasoning, etc.	MIT License
Graph Databases and Storage	Neo4j[27]	A widely used native graph database with ACID transactions and Cypher query language, suitable for highly connected data analysis.	Graph Storage, Graph Querying, Graph Algorithms, etc.	GPLv3
	JanusGraph[28]	A highly scalable graph database optimized for storing and querying large graphs with billions of vertices and edges.	Graph Storage, Gremlin Query, etc.	CC-BY-4.0
	ArangoDB[29]	A scalable graph database system with native graphs, integrated search engine, and JSON support via single query language.	Multi-Model Storage, Graph Traversal, Path Querying, etc.	BSL 1.1
	Virtuoso[30]	A hybrid relational-RDF database supporting both SPARQL and SQL, widely used for Linked Data publishing.	RDF Storage, SPARQL Query, Ontology Reasoning, etc.	GPL v2
	TigerGraph[31]	A commercial distributed parallel graph database optimized for real-time graph analytics with GSQL.	Graph Storage, Parallel Graph Computation, Real-time Querying, etc.	Proprietary
Representation Learning & Reasoning	OpenKE[32]	A sub-project of OpenSKL, providing an Open-source Knowledge Embedding toolkit for knowledge representation learning.	KG Embedding, Link Prediction, Triple Classification, etc.	MIT License
	DGL-KE[33]	A high performance, easy-to-use, and scalable package for learning large-scale knowledge graph embeddings.	KG Embedding, Large-scale Link Prediction, etc.	Apache 2.0
	PyKEEN[34]	A Python library for KG embeddings with modular design, automated hyperparameter tuning, and reproducibility guarantees.	KG Embedding, Model Evaluation, Hyperparameter Optimization, etc.	MIT License
	AmpliGraph[35]	A suite of neural machine learning models for relational Learning on knowledge graphs with supervised learning.	Generate KG embeddings, Link Prediction, Anomaly Detection, etc.	Apache 2.0
	LibKGE[36]	A PyTorch-based library for efficient training, evaluation, and hyperparameter optimization of knowledge graph embeddings.	Link Prediction, Training, Evaluation of KGE Models, etc.	MIT License
	Pykg2vec[37]	A library for learning the representation of entities and relations in Knowledge Graph with various embedding models.	KGE Model Implementations, Hyperparameters Discovery, Learned Embedding Inspecting, etc.	MIT License
Auxiliary Tools	Doccano[38]	An open-source text annotation tool with a web interface for humans to label text data efficiently.	Annotation for Text Classification, Sequence Labeling, Sequence to Sequence tasks, etc.	MIT License
	Label Studio[39]	An open source data labeling tool supporting multimodal data including text, images, audio, video, time series.	Multi-modal Data Annotation, Quality Assurance, etc.	Apache 2.0
	Gephi[40]	An award-winning open-source platform for visualizing and manipulating large graphs with interactive exploration.	Graph Visualization, Network Analysis, Community Detection, etc.	CDDL 1.0
	Cytoscape[41]	A network visualization platform originally designed for bioinformatics, now supporting general-purpose network analysis.	Graph Visualization, Attribute Integration, Topology Analysis, etc.	LGPL
	GraphGPT[42]	An experimental tool using GPT models to extract entities and relations from text and generate interactive KG visualizations.	Triple Extraction, KG Construction, Visualization, etc.	MIT License
	LlamaIndex[43]	A component for building KG indices from unstructured text, integrating triples into LLM-based retrieval pipelines.	Triple Extraction, KG Indexing, KG-based QA, etc.	MIT License

Table S3: Comparative analysis of knowledge extraction paradigms in scientific domains. This comparison highlights the trade-offs between Rule-based, Traditional NLP-based, and Large Language Model (LLM) approaches across key dimensions relevant to scientific discovery.

**Dimensions**	**Rule / Dictionary-based**	**Traditional NLP-based**	**LLM-based Generation**
Core Mechanism	**Symbolic Matching:** Relies on predefined lexicons, regular expressions, and curated ontologies.	**Statistical Pattern Recognition:** Learns probability distributions from annotated corpora.	**Generative Reasoning:** Leverages pre-trained semantic knowledge and in-context learning.
Precision vs. Recall	**High Precision / Low Recall:** Excellent for standardized entities (e.g., chemical IDs) but misses variants and novel terms.	**Balanced**: High recall for in-distribution data; precision depends on training data quality.	**Variable Precision / High Recall**: Captures implicit and long-tail relations but prone to false positives without grounding.
Annotation Needs	**None (Expert-Dependent)**: Requires domain experts to craft rules, but no labeled training data.	**High (Data-Hungry)**: Requires large-scale, high-quality annotated datasets (expensive in science).	**Low (Few-Shot)**: Can perform extraction with minimal examples via prompt engineering.
Scalability & Cost	**High Efficiency**: Low inference cost; highly scalable to millions of documents.	**Moderate**: Requires GPU for training/inference; scalable once trained.	**Low Efficiency**: High inference latency and cost; challenging for processing massive corpora.
Domain Adaptability	**Low**: Updating requires manual revision of rules/dictionaries; poor transferability.	**Low**: Suffers from catastrophic forgetting; requires retraining for new domains.	**High**: Strong zero-shot transfer capability to new scientific subfields or concepts.
Scientific Reliability	**Deterministic & Interpretable**: Fully transparent decision logic; zero hallucination risk.	**Opaque (Black-box)**: Interpretable mainly via post-hoc analysis (e.g., attention weights).	**Stochastic & Hallucination-Prone**: May generate plausible but non-existent facts; requires RAG for verification.
Complexity Handling	**Low**: Struggles with nested, discontinuous, or cross-sentence relations.	**Moderate**: Effective for sentence-level dependencies but limited in long-range reasoning.	**High**: Capable of document-level reasoning, extracting synthesis recipes, and inferring causality.
Typical Use Cases	• Extraction of high-value, well-defined core entities/relations.<br>• Verification scenarios demanding near 100% precision.<br>• Projects with rich expert knowledge but limited data/compute resources.	• High-throughput information extraction from massive literature corpora.<br>• Mature domains with stable task definitions and available labeled data.<br>• Serving as high-recall retrievers in hybrid pipelines.	• Exploratory research for discovering novel associations and generating hypotheses.<br>• Parsing highly heterogeneous texts with diverse expressions.<br>• Rapid prototyping in data-scarce domains requiring strong adaptation.

Table S4: Taxonomy of evaluation methodologies for Scientific Knowledge Graphs. This table summarizes the critical dimensions for validating SciKGs, mapping specific evaluation tasks to standard metrics and representative community benchmarks across biology, chemistry, and materials science.

Dimensions	Evaluation Focus & Objectives	Standard Metrics	Representative Benchmarks
**Extraction Quality**	**NER & Relation Extraction:** Assesses the fidelity of converting unstructured scientific text (e.g., papers) into structured triples against gold standards.	**Precision, Recall, F1:** Standard classification metrics. **Micro/Macro-F1:** Essential for handling class imbalance in sparse scientific relations.	• **BioCreative** [44]: Disease/chemical entity and relation extraction (Bio). • **CHEMDNER** [45]: chemical NER (Chem). • **MatScholar NER** [46]: Materials entity and property extraction (Material).
**Ontology Alignment**	**Entity Resolution:** Evaluates the accuracy of mapping entities from heterogeneous sources to canonical IDs (e.g., aligning mentions to PubChem/UMLS).	**Alignment Accuracy:** Proportion of correctly aligned entity pairs. **Mean Reciprocal Rank (MRR):** Rank of the correct candidate match.	• **NCBI Disease Corpus** [47]: Benchmark for disease name normalization/linking. • **UMLS** [16]: Foundational terminology for medical entity linking (Bio). • **ChEBI** [17]: Ontology for chemical entity normalization (Chem). • **Materials Project** [23]: Reference database for material compound normalization (Material).
**Graph Representation**	**Link Prediction (KGE):** Measures the graph's ability to predict missing scientific facts (e.g., drug-target interactions) based on learned embeddings.	**Hits@k (k=1, 3, 10):** Presence of true triple in top-k predictions. **MRR:** Average inverse rank of the correct tail entity.	• **OGB** [48]: Benchmark platform including large-scale biological networks. • **PharmKG** [49]: Multi-relational drug-target interaction benchmark. • **OpenBioLink** [50]: High-quality benchmark for integrative omics.
**Trustworthiness & Safety**	**Fact Verification:** Evaluates whether the graph or the LLM-generated claims grounded in the graph are factually consistent with evidence.	**Factuality Score:** Alignment with ground truth evidence. **Consistency Rate:** Logical coherence across diverse queries.	• **SciFact** [51]: Verification of scientific claims against abstracts. • **PubMedQA** [52]: Reasoning over biomedical research questions. • **HealthFC** [53]: Evidence-based fact-checking in healthcare.

Table S5: Representative SciKGs for Drug Development and Optimization

Application	Year	Publication	KG Used	Entity Types	Relation Types	Entity/Relation Counts
Drug Re-purposing	2023	Bang et al. [54]	RepoMultiKG [54]	drug, disease, gene, GO_MF, etc.	drug-disease, drug-target, drug-gene, etc.	–
	2024	Huang et al.[55]	Medical KG[55]	drug, protein, phenotype, etc.	drug-drug, disease-disease, drug-protein, etc.	124K/8.06M
	2024	Huang et al.[55]	Healx KG [55]	compound, disease, gene, etc.	compound-treats-disease, gene-associates-disease, pathway-targets-gene, etc.	–
	2025	Zhang et al.[56]	iKraph [56]	disease, gene/protein, chemical compound, e	chemical-gene, chemical-disease, disease-gene, etc.	10.7M/30.8M
	2025	Madeddu et al.[57]	VITAGRAPH [57]	compound, disease, gene, side effect, etc.	compound-compound, gene-gene,compound-disease, etc.	48K/4.00M
DDI Prediction	2020	Lin et al. [58]	Drug KG[58]	drug	drug-drug interaction	24K/669K
	2024	Wang et al. [59]	KnowDDI [59]	drug, protein, disease, etc.	drug-drug, protein-protein, drug-target, etc.	–
	2024	Xu et al. [60]	iBKH [60]	drug, protein, ATC/category, etc.	drug-target-gene, drug-transporter-gene, drug-association-pathway, etc.	129K/4.03M
	2024	Wu et al.[61]	MMDDI-KG [61]	drug, chemical, transporter, etc.	drug-chemical, drug-substructure, drug-drug interaction, etc.	–
DTI Prediction	2017	Luo et al.[62]	LTN [62]	drug, protein, disease, etc.	drug-protein, drug-drug, drug-disease, etc.	12K/1.90M
	2021	Ye et al. [63]	UniDTI-KG [63]	drug, protein, disease, etc.	drug-protein, drug-drug, drug-disease, etc,	–
	2022	Ma et al. [64]	DRKG [64]	drug, protein, disease, etc.	treatment, regulates, etc.	97K/5.87M
	2022	Feng et al. [65]	e-TSN KG [65]	drug, disease, target, etc.	target-disease, etc.	10.4M/315.8M
	2024	Hoang et al.[66]	OtterKER-KG [66]	protein, compound, drug-like bioactive, etc.	target_of, interacts_with, protein-protein, etc.	–
Virtual Screening	2019	Shang et al.[67]	Gamenet [67]	patient, clinical event, diagnosis, etc.	medication combination, drug-drug interaction, etc.	–
	2021	Bhoi et al.[68]	PerMedRec KG [68]	drug, diagnose, procedure, etc.	drug co-occurrence, drug-drug interaction, etc.	–
Drug Toxicity	2018	Zitnik et al.[69]	Decagon KG[69]	drug, protein, etc.	–	20K/5.39M
	2023	Evangelista et al.[70]	ReproTox-KG [70]	birth defect, gene, drug, etc.	birth defect-gene association, birth defect-drug association, drug-gene association, etc.	24K/580K

Table S6: Representative SciKGs for Omics Interpretation and Analysis

Application	Year	Publication	KG Used	Entity Types	Relation Types	Entity/Relation Counts
Genomics	2022	Feng et al.[71]	GenomicKB [71]	chromosome chain, coding element, non-coding element, etc.	position, regulation, expression, etc.	347M/1.36B
	2024	Mulero et al.[72]	GenoRegKG [72]	enhancer, transcription factor, disease/phenotype, TAD, gene	enhancer–gene, enhancer–transcription factor,etc.	–
	2025	Zaripova et al.[73]	PhenoKG [73]	phenotype, molecular function, cellular component, etc.	protein-protein, disease-phenotype, phenotype-phenotype, etc.	105K/1.10M
Proteomics	2022	Santos et al.[74]	CKG [74]	protein, disease, metabolite, etc.	HAS-PARENT, HAS_QUANTIFIED_ PROTEIN, etc.	20M/220M
	2022	Binder et al.[75]	PKG [75]	phenotype, endogenous ligand-drug, gene, e	expression, association, gene signatures, etc.	–
	2022	Zhang et al.[76]	ProteinKG25 [76]	gene Ontology term (molecular function, cellular component, etc.), protein sequence	protein-GO, GO-GO	612K/5.0M
	2023	Chen et al.[77]	TransPKG [77]	transporter, gene, drug, etc.	transporter-gene, transporter-drug, drug-gene,	20K/528K
	2023	Pelletier et al.[78]	CaseOLAP [78]	protein, disease, pathway, etc.	protein-disease association, protein-protein interation, protein-pathway association, etc.	27.2K/540.6K
	2024	Cheng et al.[79]	ProteinKG65 [79]	protein, GO term (molecular function, cellular component, biological process)	Protein-GO	570K/5.5M
	2024	Ni et al.[80]	Biomedical KG [80]	protein, RBP, TF, etc.	PPI, RBP__regulates, miRNA__regulates, etc.	79K/6.79M
Transcriptomic	2022	Shao et al.[81]	LRT-KG [81]	receptor, pathway, target gene, etc.	ligand-receptor interactions (LRIs), receptor activation of transcription factors (TF transcription factor regulation of target genes,	–
	2024	Cavalleri et al.[82]	RNA-KG [82]	RNA molecule, cellular component, Ontolo term, etc.	molecular interaction, regulation, association with disease/phenotype, etc.	674K/12.7M
Metabolomics	2021	Delmas et al.[83]	FORUM [83]	chemical compound & class, MeSH terms (disease, anatomy, etc.), publication	–	–
Microbiomics	2023	Sun et al.[84]	MMiKG [84]	microbiota, intermediate, disease, etc.	promote, inhibit, associated, etc.	770/1.26K
Multi-omics	2020	Jha et al.[85]	Kirchhoff's KG [85]	target (gene/protein), disease, drug, etc.	gene-gene interaction, gene-drug interaction, gene-disease association, etc.	–/459.8M
	2022	Di et al.[86]	BioTAGME KG [86]	gene, protein, disease, etc.	literature, STRING, BioTAGME, etc.	161K/40M

Table S7: Representative SciKGs for Chemical Reaction and Synthesis

Application	Year	Publication	KG Used	Entity Types	Relation Types	Entity/Relation Counts
Chemical Reaction Prediction	2016	Segler et al. [87]	MolReactKG[87]	molecule, reaction, etc.	reactant, reagent, catalyst, etc.	22.6M/–
	2021	McDermott et al. [88]	ChemReactKG [88]	chemical phase, combinations of phases	chemical reaction	Varies by system: C-Cl-Li-Mn-O-Y: 5.9K/121K, C-CI-Mn-Na-O-Y: 4.4K/46K, Fe-S-Si: 0.5K/12K, Ba-Cu-O-Y: 3.0K/34K
	2023	Zhou et al. [89]	ChemQAKG[89]	chemical species, chemical reaction, etc.	chemical reaction, chemical property, etc.	–
	2024	Xie et al.[90]	ChemSynKG [90]	reactant, product	reaction templates	820K/587K
	2024	Zhang et al.[91]	CatKG [91]	substance, element	composition, reactant-product reactant-catalyst	–/11.6K
	2025	O'Ryan et al.[92]	OrgSynKG [92]	entity, chemical, temperature, e	other compound of, work up, moiety of, etc.	19.7M/22.1M
Chemical Synthesis Path Optimizatior	2021	Jeong et al.[93]	Reaction KG[93]	compound, reaction, etc.	compound-reaction, reactant-reaction, catalyst/solvents-reaction, etc.	1.67M/–
	2022	Li et al. [94]	SA Reaction KG [94]	reactant, product, etc.	chemical reaction	2.19M/9.04M
	2022	Deagen et al.[95]	FAIRIntKG[95]	keyword, country/institution, research topic/cluster, etc.	co-citation relationships, co-occurrence relationships, etc.	8.9K/25.8K
	2025	Ma et al.[96]	MacroRetroSynKG [96]	reactant, product, intermediate, etc.	reaction condition, numerical identifier, yield, etc.	3.1K/292
Molecular Property Prediction	2022	Fang et al. [97]	ChemElemKG [97]	element, attribute, etc.	element-attribute	225/1.64K
	2022	Zhang et al.[98]	MolStruct-KG [98]	compound, drug, etc.	compound reaction, drug-drug interaction, etc.	–
	2023	Fang et al.[99]	ElementKG [99]	chemical element, functional group, etc.	inRadiusGroup1, has StateGas isPartof, etc.	201/52.1K
	2025	Jiang et al.[100]	MolKG [100]	molecule, gene/protein, disease, etc.	rotatable__bond__count, covalen unit__count, hydrogen__bond__ donor__count, etc.	185K/2.52M

Table S8: Representative SciKGs for Materials Design and Discovery

Application	Year	Publication	KG Used	Entity Types	Relation Types	Entity/Relation Counts
New Material Design	2018	Onishi et al.[101]	PSPP KG [101]	process, structure, property, etc.	binary relationship (positive/negative) between factors	2K/104
	2020	Mccusker et al.[102]	NanoMine [102]	property, material, processing method, etc.	in relation to, has value, has attribute, etc.	–
	2022	Nie et al.[103]	LIB Cathodes KG[	material	material-material	–
	2022	Aggour et al.[104]	CKG [104]	material, property, processing, microstructure, etc.	processing-microstructure, property-materi processing-material, etc.	–
	2023	Gao et al.[105]	Cu-Based Catalyst KG [105]	material, regulation method, product, etc.	–	–
	2024	Venugopal et al.[106]	MatKG [106]	material, symmetry phase label, synthesis etc.	–	70K/5.4M
	2024	Ye et al.[107]	MKG [107]	material, formula, acronym, etc.	–	163K/732K
	2024	Ghafarollahi et al.[108]	OntoBioMatKG [1	biomaterial, mechanical property, etc.	provide, possess, exhibited by, etc.	33K/49K
Material Performance Prediction	2020	Mrdjenovich et al.[109]	Propnet KG [109]	material property (band gap, density, elastic moduli), property relationship/ model, etc.	property as input to model, property as output from model, etc.	184/–
	2022	Shu et al.[110]	Grain KG [110]	grain, size attribute, orientation attribute.	grain-grain, grain-size attribute, grain-orientation.	77K/745.8K
	2023	Liu et al.[111]	Aluminum Alloy Domain KG [111]	alloy, series, material, etc.	ClassOf, subClassOf,PropertyOf, etc.	-/1.15K
	2023	Song et al.[112]	NR-KG [112]	HEA, element, processing techniques, etc.	is_structured_by, is_contained_by, is_ processed, etc.	–
	2023	Statt et al.[113]	MekG [113]	analysis, analysisDetails, collection, etc.	analysis_details, contains, collection-sample, etc.	52.3M/111.4M
	2024	Durmaz et al.[114]	MaterioMiner [114	physical quantities, materials, mechanisms, etc.	associatedwith,causeof, correlatedwith, etc.	2.19K/–
	2024	Huang et al.[115]	Element KG [115]	element, attribute, etc.	isperiodof, ismetallicityof, isstateof, etc.	–
Material Screening & Optimization	2024	Zhang et al.[116]	MGED-KG [116]	term, category, etc.	subclassof, isakindof, isrelatedto, etc.	8.9K/–
	2025	Bai et al.[117]	KG-FM [117]	property, structure, application, etc.	derived from, published in (journal), published at (date), etc.	2.53M/4.01M

Table S9: Evaluation practices for KG-LLM integration in scientific discovery. This table synthesizes current methodologies for assessing the key claimed benefits of integrating SciKGs with LLMs: improved factuality, robust reasoning, and measurable performance gains.

**Evaluation Dimension**	**Primary Objective**	**Metrics & Common Evaluation Protocols**
Factuality & Hallucination Reduction	Quantify alignment of LLM outputs with established, verifiable knowledge.	**Metrics:** Factual Accuracy, Hallucination Rate, KG-Supported Claim Ratio.
		**Protocols:** (1) Extract claims from LLM output and verify against KG triples; (2) Use of dedicated fact-checking benchmarks where answers are evidence-based.
Reasoning Robustness & Interpretability	Assess the reliability, consistency, and explainability of multi-step inferences.	**Metrics:** Multi-step QA Accuracy, Consistency Score (across related queries), Explanation Faithfulness (to KG paths).
		**Protocols:** (1) Adversarial or out-of-domain question sets to test stability; (2) Evaluation of reasoning chains for logical coherence and grounding in KG relations.
Task Performance Gain from KG Grounding	Measure the tangible improvement on a scientific task attributable to KG integration.	**Metrics:** Performance Delta (ΔAUC, ΔF1, ΔRMSE, ΔSynthesis Success Rate).
		**Protocols:** Ablation study comparing an LLM's performance *with* vs. *without* KG retrieval (RAG) on a domain-specific task dataset.

Supplementary References

[1] Michael K Gilson, Tiqing Liu, Michael Baitaluk, George Nicola, Linda Hwang, and Jenny Chong. BindingDB in 2015: A public database for medicinal chemistry, computational chemistry and systems pharmacology. *Nucleic acids research*, 44(D1):D1045–D1053, 2016.

[2] Craig Knox, Mike Wilson, Christen M Klinger, Mark Franklin, Eponine Oler, Alex Wilson, Allison Pon, Jordan Cox, Na Eun Chin, Seth A Strawbridge, et al. DrugBank 6.0: The DrugBank knowledgebase for 2024. *Nucleic acids research*, 52(D1):D1265–D1275, 2024.

[3] Allan Peter Davis, Cynthia J Grondin, Robin J Johnson, Daniela Sciaky, Jolene Wiegers, Thomas C Wiegers, and Carolyn J Mattingly. Comparative toxicogenomics database (ctd): update 2021. *Nucleic acids research*, 49(D1):D1138–D1143, 2021.

[4] Janet Piñero, Àlex Bravo, Núria Queralt-Rosinach, Alba Gutiérrez-Sacristán, Jordi Deu-Pons, Emilio Centeno, Javier García-García, Ferran Sanz, and Laura I Furlong. DisGeNET: A comprehensive platform integrating information on human disease-associated genes and variants. *Nucleic acids research*, page gkw943, 2016.

[5] Oleg Ursu, Jayme Holmes, Jeffrey Knockel, Cristian G Bologa, Jeremy J Yang, Stephen L Mathias, Stuart J Nelson, and Tudor I Oprea. DrugCentral: Online drug compendium. *Nucleic acids research*, page gkw993, 2016.

[6] Julia M Barbarino, Michelle Whirl-Carrillo, Russ B Altman, and Teri E Klein. PharmGKB: A worldwide resource for pharmacogenomic information. *Wiley Interdisciplinary Reviews: Systems Biology and Medicine*, 10(4):e1417, 2018.

[7] Michael Kuhn, Ivica Letunic, Lars Juhl Jensen, and Peer Bork. The sider database of drugs and side effects. *Nucleic acids research*, 44(D1):D1075–D1079, 2016.

[8] UniProt Consortium. UniProt: A worldwide hub of protein knowledge. *Nucleic Acids Res.*, 47(D1):D506–D15, 2019.

[9] Fiona Cunningham, James E Allen, Jamie Allen, Jorge Alvarez-Jarreta, M Ridwan Amode, Irina M Armean, Olanrewaju Austine-Orimoloye, Andrey G Azov, If Barnes, Ruth Bennett, et al. Ensembl 2022. *Nucleic acids research*, 50(D1):D988–D995, 2022.

[10] Minoru Kanehisa. The KEGG database. In *'In silico'simulation of biological processes: Novartis Foundation Symposium 247*, volume 247, pages 91–103. Wiley Online Library, 2002.

[11] Antonio Fabregat, Steven Jupe, Lisa Matthews, Konstantinos Sidiropoulos, Marc Gillespie, Phani Garapati, Robin Haw, Bijay Jassal, Florian Korninger, Bruce May, et al. The reactome pathway knowledgebase. *Nucleic acids research*, 46(D1):D649–D655, 2018.

[12] Typhaine Paysan-Lafosse, Matthias Blum, Sara Chuguransky, Tiago Grego, Beatriz Lázaro Pinto, Gustavo A Salazar, Maxwell L Bileschi, Peer Bork, Alan Bridge, Lucy Colwell, et al. Interpro in 2022. *Nucleic acids research*, 51(D1):D418–D427, 2023.

[13] Rnacentral 2021: Secondary structure integration, improved sequence search and new member databases. *Nucleic acids research*, 49(D1):D212–D220, 2021.

[14] Damian Szklarczyk, Andrea Franceschini, Stefan Wyder, Kristoffer Forslund, Davide Heller, Jaime Huerta-Cepas, Milan Simonovic, Alexander Roth, Alberto Santos, Kalliopi P Tsafou, et al. String v10: Protein–protein interaction networks, integrated over the tree of life. *Nucleic acids research*, 43(D1):D447–D452, 2015.

[15] Nicole A Vasilevsky, Nicolas A Matentzoglu, Sabrina Toro, Joseph E Flack IV, Harshad Hegde, Deepak R Unni, Gioconda F Alyea, Joanna S Amberger, Larry Babb, James P Balhoff, et al. Mondo: Unifying diseases for the world, by the world. *MedRxiv*, pages 2022–04, 2022.

[16] Olivier Bodenreider. The unified medical language system (UMLS): Integrating biomedical terminology. *Nucleic acids research*, 32(suppl_1):D267–D270, 2004.

[17] K. Degtyarenko, P. De Matos, M. Ennis, et al. ChEBI: A database and ontology for chemical entities of biological interest. *Nucleic Acids Res.*, 36(suppl_1):D344–D50, 2007.

[18] Anna Gaulton, Anne Hersey, Michał Nowotka, A Patricia Bento, Jon Chambers, David Mendez, Prudence Mutowo, Francis Atkinson, Louisa J Bellis, Elena Cibrián-Uhalte, et al. The chembl database in 2017. *Nucleic acids research*, 45(D1):D945–D954, 2017.

[19] Jonathan Goodman. Computer software review: Reaxys, 2009.

[20] S. Kim, J. Chen, T. Cheng, et al. Pubchem 2023 update. *Nucleic Acids Res.*, 51(D1):D1373–D80, 2023.

[21] Benjamin I Tingle, Khanh G Tang, Mar Castanon, John J Gutierrez, Munkhzul Khurelbaatar, Chinzorig Dandarchuluun, Yurii S Moroz, and John J Irwin. Zinc-22— a free multi-billion-scale database of tangible compounds for ligand discovery. *Journal of chemical information and modeling*, 63(4):1166–1176, 2023.

[22] Scott Kirklin, James E Saal, Bryce Meredig, Alex Thompson, Jeff W Doak, Muratahan Aykol, Stephan Rühl, and Chris Wolverton. The open quantum materials database (oqmd): Assessing the accuracy of dft formation energies. *npj Computational Materials*, 1(1):1–15, 2015.

[23] A. Jain, S. P. Ong, G. Hautier, et al. Commentary: The materials project: A materials genome approach to accelerating materials innovation. *APL Mater.*, 1(1), 2013.

[24] Ningyu Zhang, Xin Xu, Liankuan Tao, Haiyang Yu, Hongbin Ye, Shuofei Qiao, Xin Xie, Xiang Chen, Zhoubo Li, Lei Li, et al. DeepKE: A deep learning based knowledge extraction toolkit for knowledge base population. *arXiv:2201.03335*, 2022.

[25] Yujie Luo, Xiangyuan Ru, Kangwei Liu, Lin Yuan, Mengshu Sun, Ningyu Zhang, Lei Liang, Zhiqiang Zhang, Jun Zhou, Lanning Wei, et al. OneKE: A dockerized schema-guided LLM Agent-based knowledge extraction system. In *Companion Proceedings of the ACM on Web Conference 2025*, pages 2871–2874, 2025.

[26] Yuqi Zhu, Xiaohan Wang, Jing Chen, Shuofei Qiao, Yixin Ou, Yunzhi Yao, Shumin Deng, Huajun Chen, and Ningyu Zhang. LLMs for knowledge graph construction and reasoning: Recent capabilities and future opportunities. *World Wide Web*, 27(5):58, 2024.

[27] Neo4j. Neo4j official website, 2025. Software available at https://neo4j.com.

[28] Janusgraph. Janusgraph offical website, 2024. Software available at https://github.com/JanusGraph/janusgraph.

[29] ArangoDB. ArangoDB official website, 2025. Software available at https://arangodb.com.

[30] Virtuoso. Virtuoso official website, 2025. Software available at https://vos.openlinksw.com/owiki/wiki/VOS.

[31] Alin Deutsch, Yu Xu, Mingxi Wu, and Victor Lee. Tigergraph: A native mpp graph database. *arXiv:1901.08248*, 2019.

[32] Xu Han, Shulin Cao, Xin Lv, Yankai Lin, Zhiyuan Liu, Maosong Sun, and Juanzi Li. OpenKE: An open toolkit for knowledge embedding. In *Proceedings of the 2018 conference on empirical methods in natural language processing: system demonstrations*, pages 139–144, 2018.

[33] Da Zheng, Xiang Song, Chao Ma, Zeyuan Tan, Zihao Ye, Jin Dong, Hao Xiong, Zheng Zhang, and George Karypis. DGL-KE: Training knowledge graph embeddings at scale. In *Proceedings of the 43rd international ACM SIGIR conference on research and development in information retrieval*, pages 739–748, 2020.

[34] Mehdi Ali, Max Berrendorf, Charles Tapley Hoyt, Laurent Vermue, Sahand Sharifzadeh, Volker Tresp, and Jens Lehmann. PyKEEN 1.0: A python library for training and evaluating knowledge graph embeddings. *Journal of Machine Learning Research*, 22(82):1–6, 2021.

[35] Luca Costabello, Sumit Pai, CL Van, Rory McGrath, Nicholas McCarthy, and Pedro Tabacof. AmpliGraph: A library for representation learning on knowledge graphs. *Retrieved Oct*, 10:2019, 2019.

[36] Samuel Broscheit, Daniel Ruffinelli, Adrian Kochsiek, Patrick Betz, and Rainer Gemulla. LibKGE-A knowledge graph embedding library for reproducible research. In *Proceedings of the 2020 conference on empirical methods in natural language processing: system demonstrations*, pages 165–174, 2020.

[37] Shih-Yuan Yu, Sujit Rokka Chhetri, Arquimedes Canedo, Palash Goyal, and Mohammad Abdullah Al Faruque. Pykg2vec: A python library for knowledge graph embedding. *Journal of Machine Learning Research*, 22(16):1–6, 2021.

[38] Hiroki Nakayama, Takahiro Kubo, Junya Kamura, Yasufumi Taniguchi, and Xu Liang. Doccano: Text annotation tool for human, 2018. Software available from https://github.com/doccano/doccano.

[39] Maxim Tkachenko, Mikhail Malyuk, Andrey Holmanyuk, and Nikolai Liubimov. Label Studio: Data labeling software, 2020. Open source software available from https://github.com/HumanSignal/label-studio.

[40] Mathieu Bastian, Sebastien Heymann, and Mathieu Jacomy. Gephi: An open source software for exploring and manipulating networks. In *Proceedings of the international AAAI conference on web and social media*, volume 3, pages 361–362, 2009.

[41] Paul Shannon, Andrew Markiel, Owen Ozier, Nitin S Baliga, Jonathan T Wang, Daniel Ramage, Nada Amin, Benno Schwikowski, and Trey Ideker. Cytoscape: A software environment for integrated models of biomolecular interaction networks. *Genome research*, 13(11):2498–2504, 2003.

[42] Varun Shenoy. GraphGPT — build complex directed graphs from natural language, 2025.

[43] Jerry Liu. LlamaIndex, November 2022. Software available at https://github.com/jerryjliu/llama_index.

[44] Jiao Li, Yueping Sun, Robin J Johnson, Daniela Sciaky, Chih-Hsuan Wei, Robert Leaman, Allan Peter Davis, Carolyn J Mattingly, Thomas C Wiegers, and Zhiyong Lu. Biocreative v cdr task corpus: A resource for chemical disease relation extraction. *Database*, 2016, 2016.

[45] Martin Krallinger, Obdulia Rabal, Florian Leitner, Miguel Vazquez, David Salgado, Zhiyong Lu, Robert Leaman, Yanan Lu, Donghong Ji, Daniel M Lowe, et al. The chemdner corpus of chemicals and drugs and its annotation principles. *Journal of cheminformatics*, 7(Suppl 1):S2, 2015.

[46] Leigh Weston, Vahe Tshitoyan, John Dagdelen, Olga Kononova, Amalie Trewartha, Kristin A Persson, Gerbrand Ceder, and Anubhav Jain. Named entity recognition and normalization applied to large-scale information extraction from the materials science literature. *Journal of chemical information and modeling*, 59(9):3692–3702, 2019.

[47] Rezarta Islamaj Doğan, Robert Leaman, and Zhiyong Lu. Ncbi disease corpus: A resource for disease name recognition and concept normalization. *Journal of biomedical informatics*, 47:1–10, 2014.

[48] Weihua Hu, Matthias Fey, Marinka Zitnik, Yuxiao Dong, Hongyu Ren, Bowen Liu, Michele Catasta, and Jure Leskovec. Open graph benchmark: Datasets for machine learning on graphs. *Advances in neural information processing systems*, 33:22118–22133, 2020.

[49] Shuangjia Zheng, Jiahua Rao, Ying Song, Jixian Zhang, Xianglu Xiao, Evandro Fei Fang, Yuedong Yang, and Zhangming Niu. Pharmkg: a dedicated knowledge graph benchmark for bomedical data mining. *Briefings in bioinformatics*, 22(4):bbaa344, 2021.

[50] Anna Breit, Simon Ott, Asan Agibetov, and Matthias Samwald. Openbiolink: A benchmarking framework for large-scale biomedical link prediction. *Bioinformatics*, 36(13):4097–4098, 2020.

[51] David Wadden, Shanchuan Lin, Kyle Lo, Lucy Lu Wang, Madeleine van Zuylen, Arman Cohan, and Hannaneh Hajishirzi. Fact or fiction: Verifying scientific claims. *arXiv preprint arXiv:2004.14974*, 2020.

[52] Qiao Jin, Bhuwan Dhingra, Zhengping Liu, William Cohen, and Xinghua Lu. Pubmedqa: A dataset for biomedical research question answering. In *Proceedings of the 2019 conference on empirical methods in natural language processing and the 9th international joint conference on natural language processing (EMNLP-IJCNLP)*, pages 2567–2577, 2019.

[53] Juraj Vladika, Phillip Schneider, and Florian Matthes. Healthfc: Verifying health claims with evidence-based medical fact-checking. In *Proceedings of the 2024 Joint International Conference on Computational Linguistics, Language Resources and Evaluation (LREC-COLING 2024)*, pages 8095–8107, 2024.

[54] D. Bang, S. Lim, S. Lee, et al. Biomedical knowledge graph learning for drug repurposing by extending guilt-by-association to multiple layers. *Nat. Commun.*, 14(1):3570, 2023.

[55] K. Huang, P. Chandak, Q. Wang, et al. A foundation model for clinician-centered drug repurposing. *Nat. Med.*, 30(12):3601–13, 2024.

[56] Y. Zhang, X. Sui, F. Pan, et al. A comprehensive large-scale biomedical knowledge graph for ai-powered data-driven biomedical research. *Nat. Mach. Intell.*, pages 1–13, 2025.

[57] Francesco Madeddu, Lucia Testa, Gianluca De Carlo, Michele Pieroni, Andrea Mastropietro, Aris Anagnostopoulos, Paolo Tieri, and Sergio Barbarossa. VitaGraph: Building a knowledge graph for biologically relevant learning tasks. *arXiv:2505.11185*, 2025.

[58] Xuan Lin, Zhe Quan, Zhi-Jie Wang, Tengfei Ma, and Xiangxiang Zeng. KGNN: Knowledge graph neural network for drug-drug interaction prediction. In *IJCAI*, volume 380, pages 2739–2745, 2020.

[59] Yaqing Wang, Zaifei Yang, and Quanming Yao. Accurate and interpretable drug-drug interaction prediction enabled by knowledge subgraph learning. *Communications Medicine*, 4(1):59, 2024.

[60] C. Xu, K. C. Bulusu, H. Pan, et al. DDI-GPT: Explainable prediction of drug-drug interactions using large language models enhanced with knowledge graphs. *bioRxiv*, pages 2024–12, 2024.

[61] D. Wu, W. Sun, Y. He, et al. MKG-FENN: A multimodal knowledge graph fused end-to-end neural network for accurate drug–drug interaction prediction. In *Proceedings of the AAAI Conference on Artificial Intelligence*, volume 38, pages 10216–24, 2024.

[62] Y. Luo, X. Zhao, J. Zhou, et al. A network integration approach for drug-target interaction prediction and computational drug repositioning from heterogeneous information. *Nat. Commun.*, 8(1):573, 2017.

[63] Qing Ye, Chang-Yu Hsieh, Ziyi Yang, Yu Kang, Jiming Chen, Dongsheng Cao, Shibo He, and Tingjun Hou. A unified drug–target interaction prediction framework based on knowledge graph and recommendation system. *Nature communications*, 12(1):6775, 2021.

[64] Tengfei Ma, Xuan Lin, Bosheng Song, Philip S Yu, and Xiangxiang Zeng. KG-MTL: Knowledge graph enhanced multi-task learning for molecular interaction. *IEEE Transactions on Knowledge and Data Engineering*, 35(7):7068–7081, 2022.

[65] Ziyan Feng, Zihao Shen, Honglin Li, and Shiliang Li. E-TSN: An interactive visual exploration platform for target–disease knowledge mapping from literature. *Briefings in Bioinformatics*, 23(6):bbac465, 2022.

[66] Thanh Lam Hoang, Marco Luca Sbodio, Marcos Martinez Galindo, Mykhaylo Zayats, Raul Fernandez-Diaz, Victor Valls, Gabriele Picco, Cesar Berrospi, and Vanessa Lopez. Knowledge enhanced representation learning for drug discovery. In *Proceedings of the AAAI Conference on Artificial Intelligence*, volume 38, pages 10544–10552, 2024.

[67] J. Shang, C. Xiao, T. Ma, et al. Gamenet: Graph augmented memory networks for recommending medication combination. In *Proceedings of the AAAI Conference on Artificial Intelligence*, volume 33, pages 1126–33, 2019.

[68] Suman Bhoi, Mong Li Lee, Wynne Hsu, Hao Sen Andrew Fang, and Ngiap Chuan Tan. Personalizing medication recommendation with a graph-based approach. *ACM Transactions on Information Systems (TOIS)*, 40(3):1–23, 2021.

[69] Marinka Zitnik, Monica Agrawal, and Jure Leskovec. Modeling polypharmacy side effects with graph convolutional networks. *Bioinformatics*, 34(13):i457–i466, 2018.

[70] J. E. Evangelista, D. J. B. Clarke, Z. Xie, et al. Toxicology knowledge graph for structural birth defects. *Commun. Med.*, 3(1):98, 2023.

[71] F. Feng, F. Tang, Y. Gao, et al. GenomicKB: A knowledge graph for the human genome. *Nucleic Acids Res.*, 51(D1):D950–D56, 2023.

[72] J. Mulero-Hernández, V. Mironov, J. A. Miñarro-Giménez, et al. Integration of chromosome locations and functional aspects of enhancers and topologically associating domains in knowledge graphs enables versatile queries about gene regulation. *Nucleic Acids Res.*, 52(15):e69, 2024.

[73] K. Zaripova, E. Özsoy, N. Navab, et al. PhenoKG: Knowledge graph-driven gene discovery and patient insights from phenotypes alone. *arXiv:2506.13119*, 2025.

[74] A. Santos, A. R. Colaço, A. B. Nielsen, et al. A knowledge graph to interpret clinical proteomics data. *Nat. Biotechnol.*, 40(5):692–702, 2022.

[75] J. Binder, O. Ursu, C. Bologa, et al. Machine learning prediction and tau-based screening identifies potential Alzheimer's disease genes relevant to immunity. *Commun. Biol.*, 5(1):125, 2022.

[76] Ningyu Zhang, Zhen Bi, Xiaozhuan Liang, Siyuan Cheng, Haosen Hong, Shumin Deng, Jiazhang Lian, Qiang Zhang, and Huajun Chen. OntoProtein: Protein pretraining with gene ontology embedding. *arXiv:2201.11147*, 2022.

[77] Xiao-Hui Chen, Yao Ruan, Yan-Guang Liu, Xin-Ya Duan, Feng Jiang, Hao Tang, Hong-Yu Zhang, and Qing-Ye Zhang. Transporter proteins knowledge graph construction and its application in drug development. *Computational and Structural Biotechnology Journal*, 21:2973–2984, 2023.

[78] Alexander R Pelletier, Dylan Steinecke, Dibakar Sigdel, Irsyad Adam, J Harry Caufield, Vladimir Guevara-Gonzalez, Joseph Ramirez, Aarushi Verma, Kaitlyn Bali, Katherine Downs, et al. A knowledge graph approach to elucidate the role of organellar pathways in disease via biomedical reports. *Journal of Visualized Experiments*, (200), 2023.

[79] Siyuan Cheng, Xiaozhuan Liang, Zhen Bi, Ningyu Zhang, and Huajun Chen. ProteinKG65: A knowledge graph for protein science. *arXiv:2207.10080*, 2022.

[80] Shengkun Ni, Xiangtai Kong, Yingying Zhang, Zhengyang Chen, Zhaokun Wang, Zunyun Fu, Ruifeng Huo, Xiaochu Tong, Ning Qu, Xiaolong Wu, et al. Identifying compound-protein interactions with knowledge graph embedding of perturbation transcriptomics. *Cell Genomics*, 4(10), 2024.

[81] X. Shao, C. Li, H. Yang, et al. Knowledge-graph-based cell-cell communication inference for spatially resolved transcriptomic data with SpaTalk. *Nat. Commun.*, 13(1):4429, 2022.

[82] E. Cavalleri, A. Cabri, M. Soto-Gomez, et al. An ontology-based knowledge graph for representing interactions involving RNA molecules. *Sci. Data*, 11(1):906, 2024.

[83] M. Delmas, O. Filangi, N. Paulhe, et al. Building a knowledge graph from public databases and scientific literature to extract associations between chemicals and diseases. *Bioinformatics*, 37(21):3896–904, 2021.

[84] H. Sun, Z. Song, Q. Chen, et al. MMiKG: A knowledge graph-based platform for path mining of microbiota–mental diseases interactions. *Brief. Bioinform.*, 24(6):bbad340, 2023.

[85] A. Jha, Y. Khan, R. Sahay, et al. Metastatic site prediction in breast cancer using omics knowledge graph and pattern mining with Kirchhoff's law traversal. *bioRxiv*, pages 2020–07, 2020.

[86] Antonio Di Maria, Salvatore Alaimo, Lorenzo Bellomo, Fabrizio Billeci, Paolo Ferragina, Alfredo Ferro, and Alfredo Pulvirenti. BioTAGME: A comprehensive platform for biological knowledge network analysis. *Frontiers in Genetics*, 13:855739, 2022.

[87] Marwin HS Segler and Mark P Waller. Modelling chemical reasoning to predict and invent reactions. *Chemistry–A European Journal*, 23(25):6118–6128, 2017.

[88] M. J. McDermott, S. S. Dwaraknath, and K. A. Persson. A graph-based network for predicting chemical reaction pathways in solid-state materials synthesis. *Nat. Commun.*, 12(1):3097, 2021.

[89] Xiaochi Zhou, Shaocong Zhang, Mehal Agarwal, Jethro Akroyd, Sebastian Mosbach, and Markus Kraft. Marie and bert— a knowledge graph embedding based question answering system for chemistry. *ACS omega*, 8(36):33039–33057, 2023.

[90] J. Xie, Y. Wang, J. Rao, et al. Self-supervised contrastive molecular representation learning with a chemical synthesis knowledge graph. *J. Chem. Inf. Model.*, 64(6):1945–54, 2024.

[91] Z. Zhang, S. Ma, S. Zheng, et al. Semantic knowledge graph as a companion for catalyst recommendation. *Natl. Sci. Open*, 3(2):20230040, 2024.

[92] Connor O'Ryan, Kevin D Hayes, Francis G VanGessel, Ruth M Doherty, William Wilson, John Fischer, Zois Boukouvalas, and Peter W Chung. An automated approach for domain-specific knowledge graph generation— graph measures and characterization. *Journal of Chemical Information and Modeling*, 2025.

[93] J. Jeong, N. Lee, Y. Shin, et al. Intelligent generation of optimal synthetic pathways based on knowledge graph inference and retrosynthetic predictions using reaction big data. *J. Taiwan Inst. Chem. Eng.*, 130:103982, 2022.

[94] B. Li and H. Chen. Prediction of compound synthesis accessibility based on reaction knowledge graph. *Molecules*, 27(3):1039, 2022.

[95] Michael E Deagen, Jamie P McCusker, Tolulomo Fateye, Samuel Stouffer, L Cate Brinson, Deborah L McGuinness, and Linda S Schadler. Fair and interactive data graphics from a scientific knowledge graph. *Scientific Data*, 9(1):239, 2022.

[96] Q. Ma, Y. Zhou, and J. Li. Automated retrosynthesis planning of macromolecules using large language models and knowledge graphs. *Macromol. Rapid Commun.*, page 2500065, 2025.

[97] Y. Fang, Q. Zhang, H. Yang, et al. Molecular contrastive learning with chemical element knowledge graph. In *Proceedings of the AAAI Conference on Artificial Intelligence*, volume 36, pages 3968–76, 2022.

[98] Yi Zhang, Zhouhan Li, Biao Duan, Lei Qin, and Jing Peng. MKGE: Knowledge graph embedding with molecular structure information. *Computational Biology and Chemistry*, 100:107730, 2022.

[99] Y. Fang, Q. Zhang, N. Zhang, et al. Knowledge graph-enhanced molecular contrastive learning with functional prompt. *Nat. Mach. Intell.*, 5(5):542–53, 2023.

[100] P. Jiang, C. Xiao, T. Fu, et al. Bi-level contrastive learning for knowledge-enhanced molecule representations. In *Proceedings of the AAAI Conference on Artificial Intelligence*, volume 39, pages 352–60, 2025.

[101] Takeshi Onishi, Takuya Kadohira, and Ikumu Watanabe. Relation extraction with weakly supervised learning based on process-structure-property-performance reciprocity. *Science and technology of advanced materials*, 19(1):649–659, 2018.

[102] J. P. McCusker, N. Keshan, S. Rashid, M. Deagen, C. Brinson, and D. L. McGuinness. Nanomine: A knowledge graph for nanocomposite materials science. In *The Semantic Web – ISWC*, volume 12507, pages 144–59, 2020.

[103] Z. Nie, S. Zheng, Y. Liu, et al. Automating materials exploration with a semantic knowledge graph for Li-ion battery cathodes. *Adv. Funct. Mater.*, 32(26):2201437, 2022.

[104] Kareem S Aggour, Andrew Detor, Alfredo Gabaldon, Varish Mulwad, Abha Moitra, Paul Cuddihy, and Vijay S Kumar. Compound knowledge graph-enabled ai assistant for accelerated materials discovery. *Integrating Materials and Manufacturing Innovation*, 11(4):467–478, 2022.

[105] Y. Gao, L. Wang, X. Chen, et al. Revisiting electrocatalyst design by a knowledge graph of Cu-based catalysts for CO2 reduction. *ACS Catal.*, 13(13):8525–34, 2023.

[106] V. Venugopal and E. Olivetti. MatKG: An autonomously generated knowledge graph in material science. *Sci. Data*, 11(1):217, 2024.

[107] Y. Ye, J. Ren, S. Wang, et al. Construction and application of materials knowledge graph in multidisciplinary materials science via large language model. *Adv. Neural Inf. Process. Syst.*, 37:56878–97, 2024.

[108] A. Ghafarollahi and M. J. Buehler. SciAgents: Automating scientific discovery through bioinspired multi-agent intelligent graph reasoning. *Adv. Mater.*, page 2413523, 2024.

[109] D. Mrdjenovich, M. K. Horton, J. H. Montoya, et al. PropNet: A knowledge graph for materials science. *Matter*, 2(2):464–80, 2020.

[110] Chao Shu, Junjie He, Guangjie Xue, and Cheng Xie. Grain knowledge graph representation learning: A new paradigm for microstructure-property prediction. *Crystals*, 12(2):280, 2022.

[111] Jian Liu and Quan Qian. Reinforcement learning-based knowledge graph reasoning for aluminum alloy applications. *Computational Materials Science*, 221:112075, 2023.

[112] Guangxuan Song, Dongmei Fu, Zhongwei Qiu, Zijiang Yang, Jiaxin Dai, Lingwei Ma, and Dawei Zhang. Bridging the semantic-numerical gap: A numerical reasoning method of cross-modal knowledge graph for material property prediction. *arXiv:2312.09744*, 2023.

[113] Michael J Statt, Brian A Rohr, Dan Guevarra, Ja'Nya Breeden, Santosh K Suram, and John M Gregoire. The materials experiment knowledge graph. *Digital Discovery*, 2(4):909–914, 2023.

[114] Ali Riza Durmaz, Akhil Thomas, Lokesh Mishra, Rachana Niranjan Murthy, and Thomas Straub. An ontology-based text mining dataset for extraction of process-structure-property entities. *Scientific data*, 11(1):1112, 2024.

[115] C. Huang, C. Chen, L. Shi, et al. Material property prediction with element attribute knowledge graphs and multimodal representation learning. *arXiv:2411.08414*, 2024.

[116] Y. Zhang, F. Chen, Z. Liu, et al. A materials terminology knowledge graph automatically constructed from text corpus. *Sci. Data*, 11(1):600, 2024.

[117] X. Bai, S. He, Y. Li, et al. Construction of a knowledge graph for framework material enabled by large language models and its application. *npj Comput. Mater.*, 11(1):51, 2025.
